# Engineering multiple levels of specificity in an RNA viral vector

**DOI:** 10.1038/s41467-026-71033-7

**Published:** 2026-04-02

**Authors:** Lucy S. Chong, Jeewoo Kang, Michaela H. Ince, Matthew S. Kim, Xiaojing J. Gao, Michael B. Elowitz

**Affiliations:** 1 Howard Hughes Medical Institute, Division of Biology and Bioengineering, Broad Center, California Institute of Technology, Pasadena, CA USA; 2 Neurosciences Interdepartmental Program, Stanford University, Stanford, CA USA; 3 Department of Biochemistry and Biophysics, University of California, San Francisco, San Francisco, CA USA; 4 Cell Design Institute, University of California, San Francisco, San Francisco, CA USA; 5 Department of Chemical Engineering, Stanford University, Stanford, CA USA

**Keywords:** Biological techniques, Synthetic biology

## Abstract

Engineered molecular circuits encoded in RNA can act as programmable therapeutics that sense cellular states and elicit precise responses within diseased cells. However, their application depends critically on systems for delivering circuits into cells. Here, we engineer a model delivery system based on the rabies virus that incorporates multiple levels of control over the viral life cycle and cargo. We demonstrate controlled release of viral vectors from sender cells, conditional entry into target cells based on cell-surface proteins, restricted viral replication governed by intracellular protein content, and an escaper-resistant mechanism for viral elimination with drugs. In parallel, we integrate RNA-sensing and protease-controlled circuits to regulate cargo expression and activity at post-transcriptional and post-translational levels. Together, these strategies illustrate how viral and protein engineering can establish multi-level control at both the viral and cargo levels to facilitate specificity in future therapeutic RNA delivery systems.

## Abstract (web summary)

Engineered molecular circuits encoded in RNA can act as programmable therapeutics that sense cellular states and elicit precise responses within diseased cells. Here, the authors introduce a model delivery system with layered control of targeted infection, conditional replication, drug-inducible viral clearance, and molecular circuit-based cargo regulation, demonstrating a versatile platform for precise RNA viral vector delivery.

## Introduction

The ability to deliver designed nucleic acids to target cell types would open powerful possibilities for basic research and therapeutic applications. Improved delivery capabilities could enable new generations of gene therapy that take advantage of advances in synthetic biology to provide increased specificity and control. In fact, synthetic biologists have now developed a broad range of biological circuit designs that sense and respond to endogenous cellular states^1–4^. Recent advances in synthetic biology have enabled molecular circuits based on protein-DNA interaction^5^, regulatory RNAs^6^, and protein-level designs^7–9^, as well as combinations of these modalities^10,11^. While circuit engineering has progressed enormously, our limited ability to deliver circuits to cells in vivo has prevented their therapeutic use. A specific, effective, and controllable method to transfer circuits into target cell types could provide a foundation for the future development of circuits as therapeutics.

Viruses possess powerful capabilities as delivery systems. Viruses can preferentially infect specific cell types, and then replicate intracellularly to high levels, enabling strong expression of virally encoded proteins, potentially including engineered cargo genes. Researchers have therefore engineered diverse classes of DNA and RNA viruses for gene therapy^12,13^ and cancer therapeutic^14^ applications. Among these, RNA riboviruses^12,13,15^ (RNA viruses, excluding retroviruses) offer unique advantages, since they remain at the RNA level, avoiding integration into the host genome and potential mutagenesis of the host. More specifically, viruses in the order *Mononegavirales* have compact, well-studied genomes that offer multiple avenues for engineering control, can support high-level expression of protein components, and exhibit relatively high genomic stability^16^. Notable examples of engineered viruses from this order include vesicular stomatitis virus (VSV) for oncolytic viral therapies^17^, Sendai virus for stem cell reprogramming^18^, and rabies virus for synaptic tracing^19^.

Rabies virus could provide an alternative platform to integrate multiple levels of external and internal control with minimal risk of host genome integration. The rabies virus has been well-characterized and extensively used in neurobiology contexts. Furthermore, its ability to spread can be limited and controlled by deleting the glycoprotein, G, from the viral genome and supplying it in the host cell in *trans*^20^. Such G-deleted rabies viruses can also be pseudotyped to target specific cell surface proteins^21^. Additionally, researchers have explored the possibility of making rabies infection transient through the modification or deletion of an essential viral protein to address cytotoxicity concerns^22–24^.

Nevertheless, transforming the rabies virus into a practical circuit delivery system requires integrating existing control mechanisms into a single platform and developing additional capabilities that together allow independent control of multiple stages of the viral life cycle. To limit viral production in space and time, this system should enable control of viral exit from virus-producing cells based on inducers or environmental signals. To restrict the virus to specific targeted cell types, it should also limit viral entry to cells expressing selected cell-surface antigens and make viral replication conditional on the expression of intracellular proteins. For external control, the overall system should be controllable with a well-characterized and safe small-molecule drug. Finally, to maximize safety and minimize unintended toxicity from the virus itself, viral infection should be reversible through a control system that is resistant to escape mutations.

In addition to viral life cycle controls, the platform can incorporate sense-and-respond mechanisms to control cargo expression based on cell types and states. By operating intracellularly, these synthetic circuits can interface with not only surface antigens but also transcript levels, protein activities, and signaling dynamics, thereby directly interrogating or modulating the core pathways that drive cellular states. For example, the live RNA sensing using adenosine deaminases acting on RNA (RADAR) system^25^ enables programmable detection of specific transcripts to selectively express proteins^26,27^, while the Retained Endoplasmic Cleavable Secretion (RELEASE) system^28^ provides an interface between intracellular signals and the controlled secretion or display of proteins^29^. Most recently, engineered protein circuits have been demonstrated to specifically and sensitively kill Ras-mutant cancer cells^4^.

**Fig. 1:**
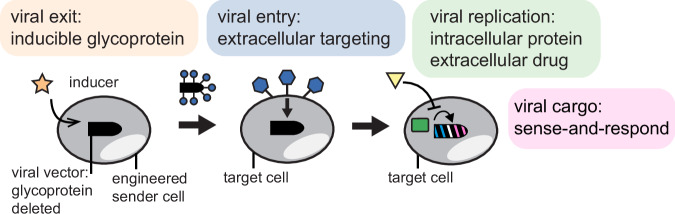
A RNA viral circuit delivery system with distinct levels of control. Sender cells (left) would be engineered to secrete the virus under the control of an inducer (orange star). Produced viruses would be packaged using a heterologous glycoprotein (pseudotyped, blue circles) to selectively infect target cells (right) expressing a desired surface antigen (blue hexagons). Once inside the target cell, viral replication would be conditional on the presence of an intracellular protein (green square). For safety, it should also be possible to suppress viral replication and eliminate the virus using a drug (yellow triangle). Additionally, the delivered viral cargo can sense-and-respond to RNA and protein content to control cargo output (blue and pink virus).

Here, we set out to engineer control of viral release, entry, replication, and cargo production by combining rabies virus control strategies with sense and response circuits (Fig. 1). These results integrate multiple levels of control and provide a framework for RNA viral vector platform engineering to support therapeutic synthetic biology and gene therapy.

## Results

### Doxycycline-inducible expression of glycoprotein controls viral exit from sender cells

**Fig. 2:**
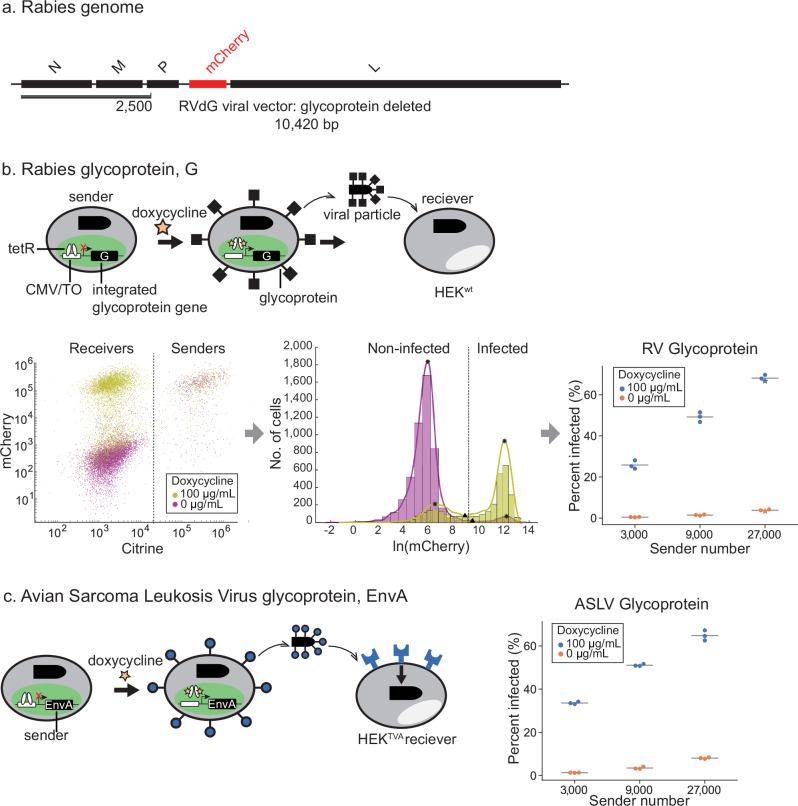
Doxycycline-inducible expression of glycoprotein controls viral exit from sender cells. **a** Of the five proteins encoded by the rabies viral genome, the glycoprotein (G) gene was replaced with mCherry (red) to generate RVdG virus. RVdG was reconstituted and propagated in producer cells that provide G in trans from the cellular genome. **b** Top: sender cell lines with a single copy of genomically integrated, doxycycline-inducible G for the controlled secretion of RVdG. The senders were also labeled with constitutively expressed Citrine. Wildtype HEK293 (HEK^wt^) target cells were co-cultured with sender cells to quantify the release of infection-competent RVdG. Left: flow cytometry of sender and receiver co-culture in the presence or absence of doxycycline. Vertical line separates receiver and sender subpopulations. Middle: distribution of mCherry signal in HEK^wt^ in the presence or absence of doxycycline (after gating out Citrine positive sender cells) measured with flow cytometry. Vertical line indicates the mean local minima used to threshold between infected and weakly infected/non-infected cells. The percent infected metric reflects the fraction of cells with signal above the threshold (Supplementary Fig. 1, middle; see Methods). The data points in the swarmplot corresponding to the examples are indicated with stars. Swarmplots are used in subsequent panels. Right: percent of target cells infected in the presence or absence of doxycycline under varying numbers of sender cells. **c** Replacing the G protein in (**b**) with a sarcoma leukosis virus glycoprotein gene (EnvA) under a doxycycline-inducible promoter. Right: percent of TVA-displaying target cell (HEK^TVA^) infected in the presence or absence of doxycycline under varying numbers of sender cells. In (**b**) and (**c**), each dot represents one biological replicate, and the horizontal lines indicate the mean of the data in each group (*n* = 3).

We first engineered a controllable ‘sender’ cell line that releases viral particles in response to external induction. This mimics a therapeutic scenario where engineered immune cells transmit viral vectors to neighboring disease cells in response to disease state signals. The system takes advantage of the well-characterized paradigm of glycoprotein (G) trans-complementation, in which the G gene is removed from the viral genome and instead expressed in the host cell line to permit single-step infection^18^ (Fig. 2a). We first deleted the native viral G gene, replacing it with mCherry for visualization (RVdG). We then incorporated a single copy of the G transgene in the host genome using the Flp-In system in HEK293 cells (Fig. 2a, b, Methods). The ectopic G gene was controlled by a CMV-TO promoter that was normally repressed by constitutively expressed TetR but could be readily induced by the addition of doxycycline (dox) or 4-epi-Tc (Fig. 2a, b, Methods). This design could be adapted to allow conditional regulation of G protein expression by other transcriptional inputs, including natural or synthetic signaling pathways^30–32^. We also stably incorporated a nuclear-localized Citrine (H2B-Citrine) as a marker.

To validate the inducibility of viral release, we co-cultured a minority of RVdG-infected sender cells with a majority of HEK293 target cells (HEK^wt^), in the presence or absence of doxycycline. After 3 days, we measured the level of mCherry signal in target cells using flow cytometry. Target cell mCherry intensities exhibited a bimodal distribution, consistent with the presence of uninfected and infected cell populations (Fig. 2b).

Using this system, we next asked how the rate of target cell infection depended on doxycycline induction and sender cell density. The clean separation between the mCherry distributions allowed quantification of infected and uninfected population sizes in different conditions (Fig. 2b, right, Supplementary Methods). As expected, infection rate depended strongly, though not absolutely, on the presence of doxycycline, with low basal levels of infection likely due to leaky expression of G from its CMV-based TetR-repressible promoter (Fig. 2b, right). Infection also exhibited a dose-dependent, but sub-linear, increase with sender cell fraction (Fig. 2b, right).

This approach can be extended to enable controlled secretion of pseudotyped viruses, in which the native viral glycoprotein is replaced with a distinct viral glycoprotein conferring different tropism^33^. For example, the well-characterized EnvA glycoprotein from the avian sarcoma leukosis virus (ASLV) binds specifically to the avian TVA protein, and abolishes rabies’ original tropism for mammalian cells^21,34^. As a demonstration, we engineered an EnvA-pseudotyped sender cell line and a cognate target cell line expressing TVA (Fig. 2c, left). As expected, viral infection was strongly dox-dependent and increased in a dose-dependent manner with sender cell number (Fig. 2c, right). Together, these results indicate that inducible glycoprotein expression enables external regulation of viral secretion for viruses both with wild-type G and pseudotyped with EnvA.

### Pseudotyping and bridge proteins control viral entry

**Fig. 3:**
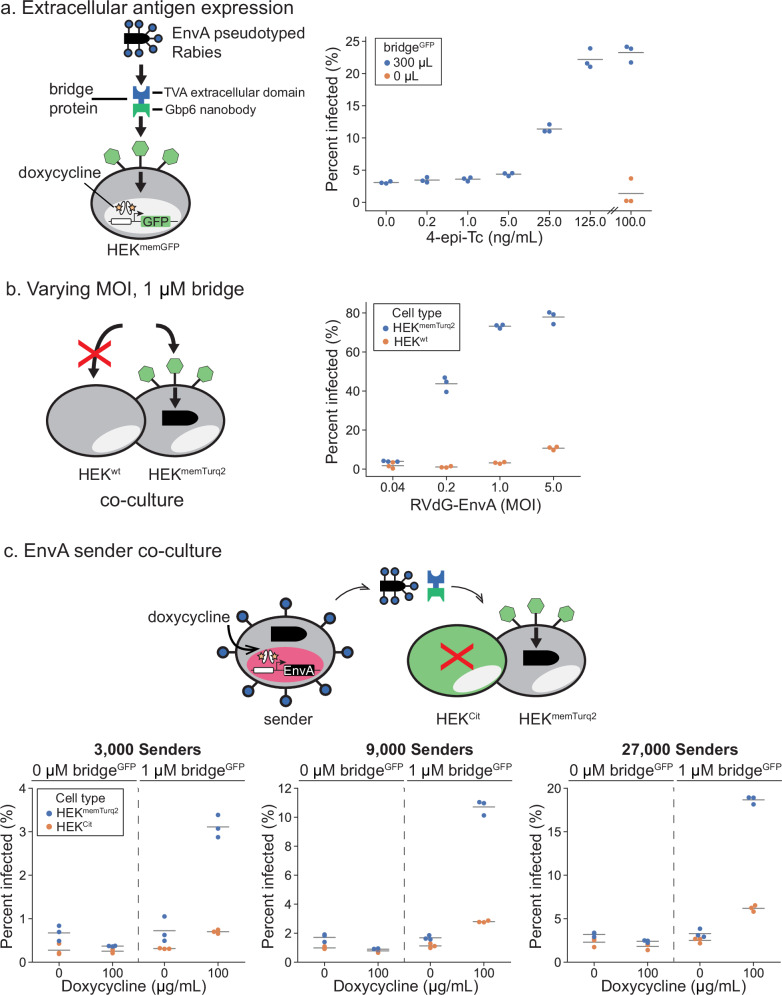
Pseudotyping and bridge proteins control viral entry. **a** Left: schematic of the bivalent bridge protein directing EnvA-pseudotyped rabies (RVdG-EnvA) to infect GFP-displaying target cells (HEK^memGFP^). Recombinantly expressed bridge protein contains the TVA receptor extracellular domain and Gbp6 nanobody (bridge^GFP^). Right: percent of HEK^memGFP^ infected under increasing concentrations of 4-epi-Tc using 1 MOI RVdG-EnvA and 250 μL of bridge^GFP^-conditioned media. **b** Constant bridge^GFP^ and varying concentrations of RVdG-EnvA administered to a mixed population of HEK^wt^ and HEK^memTurq2^ preferentially infected HEK^memTurq2^ cells. **c** Top: co-culture of doxycycline-inducible EnvA sender cells co-expressing IFP with target cells HEK^memGFP^ and non-target cells HEK^Cit^. Bottom: percent of target and non-target cells infected in the presence or absence of doxycycline and bridge protein under varying sender numbers. In (**a**–**c**), each dot represents one biological replicate and the horizontal lines indicate the mean of the data in each group (*n* = 3).

Pseudotyping opens up the possibility of targeting engineered rabies viruses to specific mammalian cell types based on their expression of surface proteins^35^. To achieve this, we took inspiration from previous efforts of targeting viral infection to specific cells based on their surface proteins^36–39^. We engineered a bivalent bridge protein, consisting of the TVA extracellular domain fused to a nanobody that recognizes GFP (Fig. 3a, left). In this modular scheme, viral entry requires both the bridge protein and expression of GFP on the surface of the target cell.

We purchased EnvA-pseudotyped rabies virus (RVdG-EnvA), engineered HEK293 derivatives inducibly expressing GFP or Turq2 (which exhibits shifted spectra but is still recognized by the GFP nanobody) on the cell surface as target cells with the addition of 4-epi-Tc (HEK^memGFP^, HEK^memTurq2^ Fig. 3a, left), and also engineered the bridge protein, denoted bridge^GFP^. To characterize the specificity of the resulting system, we added RVdG-EnvA to co-cultured parental HEK^wt^ cells and target HEK^memGFP^ cells, either with or without the bridge^GFP^. As expected, infection was strongly enhanced by the combination of surface GFP/Turq2 expression and the addition of bridge^GFP^ (Fig. 3a, right, Supplementary Fig. 1a). Infection of HEK^memGFP^ cells was not detectable without bridge^GFP^ at a multiplicity of infection (MOI) of 1. At higher MOIs of 5, some non-specific infection of HEK^wt^ and HEK^memGFP^ cells did occur without bridge protein (Fig. 3b). However, the infection rate of HEK^memGFP^ was 8-fold higher with the bridge protein than without it, even at this high MOI value. Furthermore, even at a more modest MOI of ~1, infection remained dependent on the bridge protein (Supplementary Fig. 1b). The pseudotyping bridge protein strategy thus provided strong infection specificity contingent upon surface proteins. In principle, the same design could be adapted to target natural cell types based on cell surface markers by replacing the GFP-targeting nanobody with a corresponding binding domain.

We next sought to combine inducible secretion with bridge-dependent infection. We co-cultured RVdG-infected EnvA sender cells together with both target HEK^memTurq2^ and non-target HEK^Cit^ cells. We then analyzed the relative infection rates of target and non-target cells across different viral secretion rates and numbers of sender cells with or without the bridge protein. As expected, infection strongly depended on the level of induction of the EnvA senders, the presence of bridge^GFP^, and the expression of surface antigen, making infection simultaneously dependent on all three factors (Fig. 3c).

### Viral replication can be controlled by an intracellular protein

The ability to control or condition viral replication on intracellular proteins and external inducers would enable cell-type-specific control of viral infection. We therefore sought to engineer a replication control system that could be sensitive to the presence of a specific intracellular target protein, such as a cell type marker or a protein expressed in specific states (e.g., active proliferation), as well as external small molecule inducers. To connect protein sensing to viral replication in a modular fashion, one needs to design a protein whose activity is both required for viral replication and also dependent on the presence of an inducer and/or intracellular target protein. To achieve this, we identified essential viral proteins that can be regulated by the attachment of conditional degradation domains (degrons). The DHFR degradation domain (degron) destabilizes attached proteins but can be inhibited by the drug trimethoprim (TMP)^40^. To identify sensitive sites, we fused DHFR with each essential protein and screened for locations that permitted viral replication in the presence of TMP. The C-terminus of the viral P protein provided a suitable site for regulation. It was sensitive to DHFR incorporation, but this effect could be blocked by TMP. We then introduced a TEV cleavage site between the P protein and the degron (P-DHFR), so that the virus (RVdG-P-DHFR) can be recovered from a cell line stably expressing the TEV protease without the need for TMP (Supplementary Fig. 2a).

**Fig. 4:**
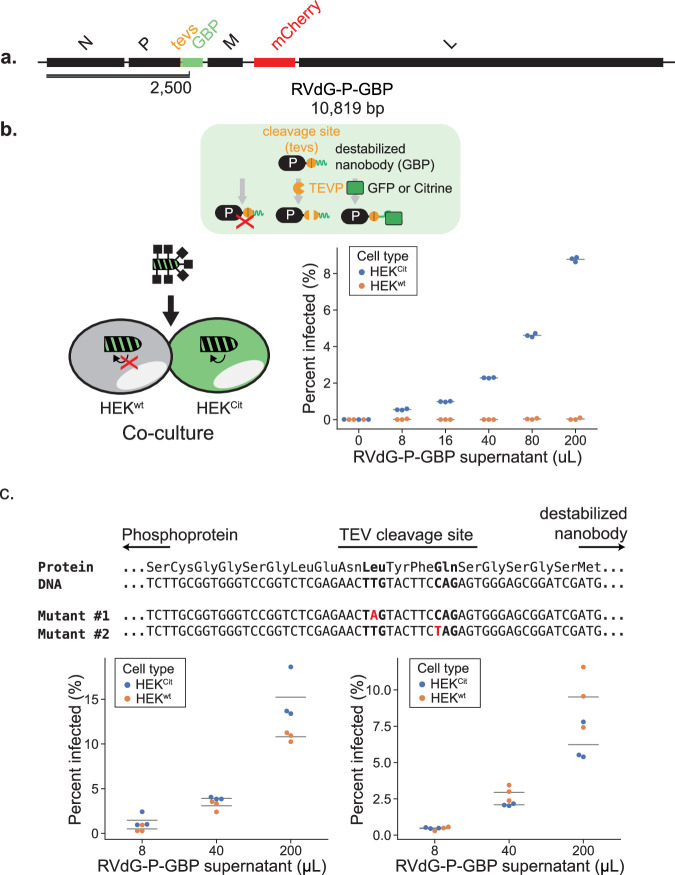
Viral replication is controlled by an intracellular protein. **a** Recombinant rabies genome, RVdG-P-GBP. **b** Tagging the phosphoprotein (P) with a degron controls viral replication. Top: schematic of a P protein (black rectangle) tagged with a destabilized nanobody, GBP (green wavy line), and an intervening TEV protease cleavage site (orange circle). P is stabilized when GFP is removed by TEV protease or stabilized when bound to GFP, thus permitting viral replication. Bottom left: in a mixed population of HEK^wt^ and HEK293 with cytoplasmic expression of Citrine (HEK^Cit^), RVdG-P-GBP will preferentially replicate in HEK^Cit^. Right: increasing concentration of RVdG-P-GBP infects HEK^Cit^ but not HEK^wt^. **c** Top: the P-GBP design exhibits evolutionary escape. Sanger sequencing of the junction region between P and GBP of two escape mutants identified two distinct nonsense mutations. Red indicates single nucleotide mutations and bold indicates mutated codons. Bottom: infection of a mixed population of HEK^wt^ and HEK^Cit^ using mutant RVdG-PGBP showed diminished discrimination. In (**b**) and (**c**), each dot represents one biological replicate, and the horizontal lines indicate the mean of data in each group (*n* = 3).

To enable regulation by endogenous proteins, we took advantage of an engineered unstable nanobody that is stabilized by binding to its target antigen^41^. We replaced the DHFR in RVdG-P-DHFR with the destabilized GFP nanobody, denoted GBP (RVdG-P-GBP, Fig. 4a, b top). To test whether viral replication was indeed conditional on expression of intracellular GFP, we used RVdG-P-GBP to infect a co-culture of Citrine-expressing HEK^Cit^ cells and non-expressing parental HEK^wt^ cells (Fig. 4b, bottom left). As measured by mCherry expression, the citrine-positive cells were infected at high rates (Fig. 4a, bottom right), while infection levels in citrine-negative cells were indistinguishable from background (Fig. 4a, bottom right). This result indicates that rabies virus replication can be made dependent on the presence of an unrelated intracellular protein (GFP).

Despite the success of the P-degron strategy, conditionality was diminished after ~1 month of continuous viral passaging in the producer cells (Fig. 4c, bottom, Supplementary Table 2, see Methods). Given the error rate of rabies RNA-dependent RNA polymerase^41^, we reasoned that this loss of function could arise from mutations that rescue P function in the absence of the target protein. Indeed, when we sequenced the P-degron junction in the RVdG-P-GBP viral genome, we consistently found nonsense mutations that truncate the protein before the degron is translated (Fig. 4c, top, Supplementary Fig. 3c), bypassing regulation. Similar escape mutants have been independently reported in other work^42^, provoking a need for engineering escaper resistance.

### An external drug inhibits viral replication and permits viral removal

**Fig. 5:**
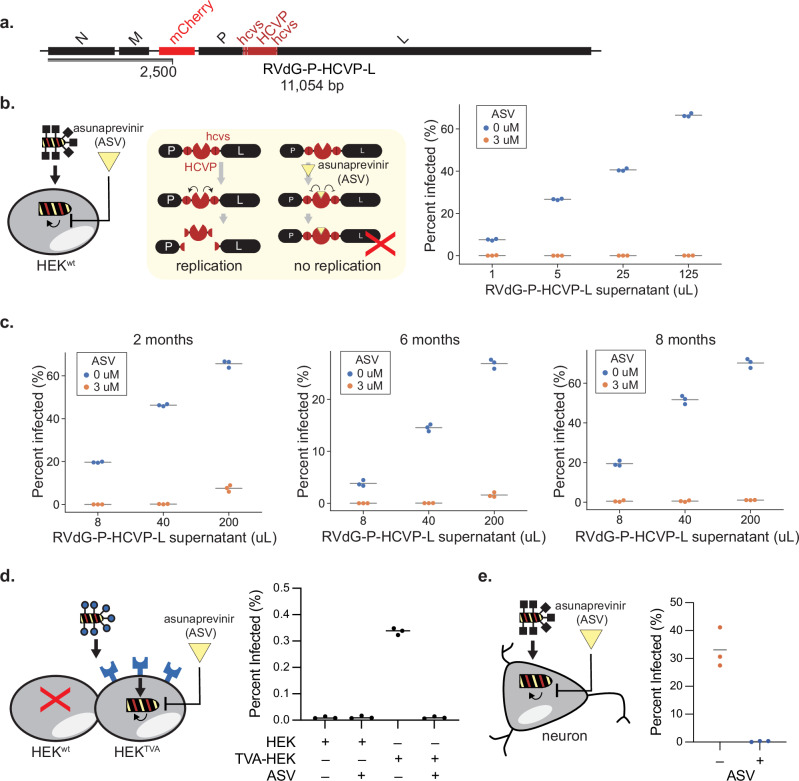
An external drug inhibits viral replication and permits viral removal. **a** Design for recombinant rabies viruses sensitive to drug inhibition, RVdG-P-HCVP-L. **b** Asunaprevir (ASV) inhibits viral replication. Left: a Hepatitis C Virus protease (HCVP, red pac-man) flanked by its cleavage sites (hcvs, red circles) was inserted between the P and L proteins to create a P-HCVP-L fusion. HCVP cleavage of the flanking cleavage sites separates P and L to permit viral replication. Addition of ASV, an HCVP inhibitor, results in non-functional P-HCVP-L fusion proteins and inhibits RVdG-P-HCVP-L viral replication. **c** RVdG-P-HCVP-L exhibited enhanced evolutionary stability. RVdG-P-HCVP-L collected from 2, 6, and 8 months of continuous passage were tested on HEK^wt^. Regulation by ASV was maintained. **d** EnvA pseudotyped RVdG-P-HCVP-L specifically infected TVA-HEK and was ASV sensitive. **e** ASV addition disrupted RVdG-P-HCVP-L replication in rat cortical neurons. In (**b**–**e**), each dot represents one biological replicate, and the horizontal lines indicate the mean of data in each group (*n* = 3). Quantification by flow cytometry in (**b**–**d**). Microscopy and image analysis in (**e**).

To reduce selection pressure for escape mutants and improve escaper resistance in our intended use case (drug-controlled reversal of established infection) we inserted an HCV protease (HCVP) flanked by its cognate cleavage sites^43–46^ between P and L (RVdG-P-HCVP-L, Fig. 5a). In this design, HCVP normally acts to cleave both sites, separating the P and L proteins and allowing them to function (Fig. 5b, left). Critically, however, the drug asunaprevir (ASV) blocks HCV protease activity^47^, leaving P and L tethered unproductively together, disrupting viral replication (Fig. 5b, left). This strategy disfavors selection for escaper mutants in two ways. First, contrary to the previous strategy, here ASV is absent when the virus is being passaged or infection is being established (see the curing experiment below), relieving the selection pressure for HCVP to lose sensitivity to ASV inhibition. Second, point mutations or deletions, the most common modes of mutations in rabies virus, should not permit the transcription and translation of L as a protein separate from P.

We passaged the RVdG-P-HCVP-L virus in producer cell lines for 8 months in the absence of ASV, periodically assaying viral sensitivity to ASV. This long-term passaging did not detectably diminish ASV sensitivity (Fig. 5c), suggesting that, compared to the previous vector, this cut-out design limits the emergence of escape mutants and extends the timescales over which pharmaceutical regulation can be maintained.

To evaluate the feasibility of combining controls across different levels, we tested pseudotyped EnvA-RVdG-P-HCVP-L in HEK293 and TVA-expressing HEK293 cells in the presence or absence of ASV. mCherry expression was detected only in TVA-HEK cells in the absence of ASV, indicating simultaneous, orthogonal control of viral replication and entry (Fig. 5d). The low infection percentages reflect the low MOI resulting from the inherently low yields of EnvA-pseudotyped virus preparations. Finally, to validate viral controls in the intended cellular context, we showed that the P-HCVP-L design was also effective in primary rat cortical neurons (Supplementary Fig. 5), where ASV addition at the time of infection nearly abolished viral replication (Fig. 5e).

**Fig. 6:**
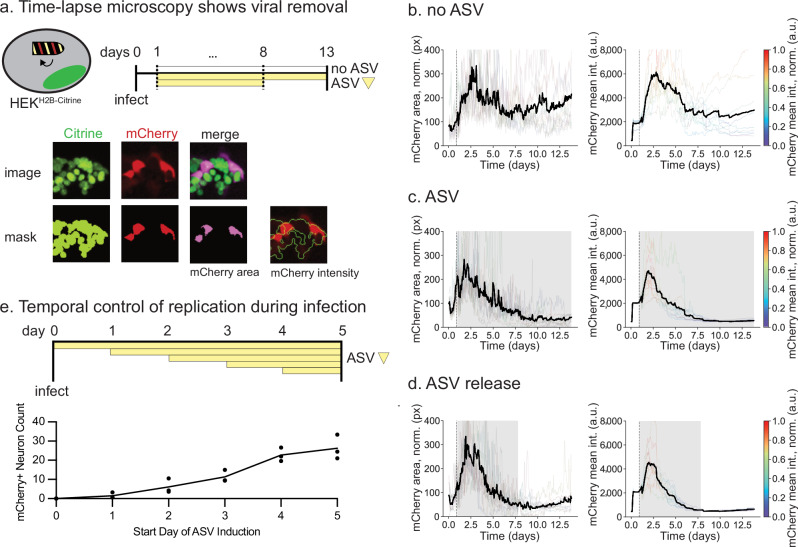
Time-lapse microscopy shows viral removal from established infections. **a** Top: timeline of infection with no ASV (top blank bar), continuous ASV (middle yellow bar), or ASV-then-release (bottom shorter yellow bar). Bottom: example of image processing. A binary mask was created based on the Citrine signal. The mCherry area shows the overlay between the mCherry mask and the Citrine mask. Note that not all mCherry+ areas are Citrine+, because the tagged H2B-Citrine is localized in the nucleus, while mCherry does not have a localization tag. The mean intensity of the mCherry signal in the Citrine mask was quantified. HEK^H2B-Citrine^ were infected for 24 hours with RVdG-P-HCVP-L (dotted line) and then cultured in media containing no ASV (**b**, no ASV), continuous ASV (**c**, ASV), or ASV for 7 days and then for 5 days in media containing no ASV (**d**, ASV release). Grey shading indicates the presence of ASV in media. Transparent and black lines, respectively, represent traces from individual movies and the mean of those traces. The fraction of infected cells as indicated by “mCherry area, norm (px)” (**b**–**d**, left column) was calculated as the fraction of mCherry+ pixels within the H2B-Citrine+ mask. Individual traces for mCherry intensity are color-scaled from purple to red, where mCherry intensity values are normalized by the maximum mCherry intensity within each trace. The slight increases at late times in the mean mCherry intensity traces represent cellular autofluorescence. In order to display all mean traces on the same y-scale, the tops of some individual traces are cut off. For complete traces, see Supplementary Fig. 3. **e** Top: timeline of RVdG-P-HCVP-L infection (d0) with ASV induction at time of infection (top yellow bar) and varying days d1 - d4 (middle yellow bars). No ASV added on day of imaging d5 (bottom yellow bar). Bottom: data represents the mean number of mCherry+ neurons from 5 field images for each replicate (*n* = 3) at the time of ASV induction. Arbitrary units: a.u.

So far, we have focused on controlling the establishment of infection. However, for many applications, it will be crucial to terminate a productive infection after the viral vector and the encoded circuit perform their function. Therefore, we tested the ability of ASV to cure an established infection by the engineered virus. We used time-lapse microscopy to follow the dynamics of infection in the same population of cells over time before and after ASV addition (Fig. 6a).

To evaluate viral clearance, we incubated HEK293 cells that express a constitutive H2B-Citrine (HEK^H2B-Citrine^) with viral particles, and quantified mCherry signal as a surrogate for the level of infection using two similarly behaving metrics (Fig. 6a, see Methods for details). After one day, we observed increased mCherry signal (Fig. 6b–d), consistent with viral infection and replication. This signal remained high for more than ∼13 days (Fig. 5b, Supplementary Movie 1, left). In a parallel experiment, we added ASV to the media after one day (Fig. 6c). This treatment led to a decay in the mCherry signal over the following 7 days, until no signal could be detected above background (Fig. 6c, Supplementary Movie 1, center). Thus, ASV addition appeared to successfully terminate viral protein expression.

Nevertheless, in principle, low levels of virus could remain after ASV treatment and resume replication after ASV removal. We therefore sought to more rigorously determine whether transient addition of ASV could permanently cure an established infection of the engineered virus. First, in a preliminary experiment, we treated infected cells with two concentrations of ASV for 6 days (see Methods) and then removed the drug for 3 days. We observed no viral re-emergence in all but one field of view. This result suggested the potential for further improvement through repeated and prolonged ASV dosing (Supplementary Fig. 4a and 4b, Supplementary Movie 2). We therefore extended the period of ASV exposure an additional overnight period, during which we carried out daily media changes. We then washed out the drug and continued to monitor the culture for 5 more days (Fig. 6d, Supplementary Movie 1, right), which is more than sufficient time for intracellular amplification. We observed no re-emergence of infection during this time across all ten fields of cells (Fig. 6d). These results suggest that infections of the engineered virus could be successfully reversed by transient ASV addition in the overwhelming majority of cells.

We next aimed to systematically examine the effect of inhibition duration on the extent of viral infection. Rat cortical neurons were infected with viral particles, and ASV was administered either at the time of infection, 1 − 4 days post-infection, or not at all (Fig. 6e, top). These experiments suggest that the extent of infection can be temporally regulated by adjusting the interval between viral entry and termination of replication (Fig. 6e, bottom).

### Two circuits control viral cargo production at transcript and protein levels

**Fig. 7:**
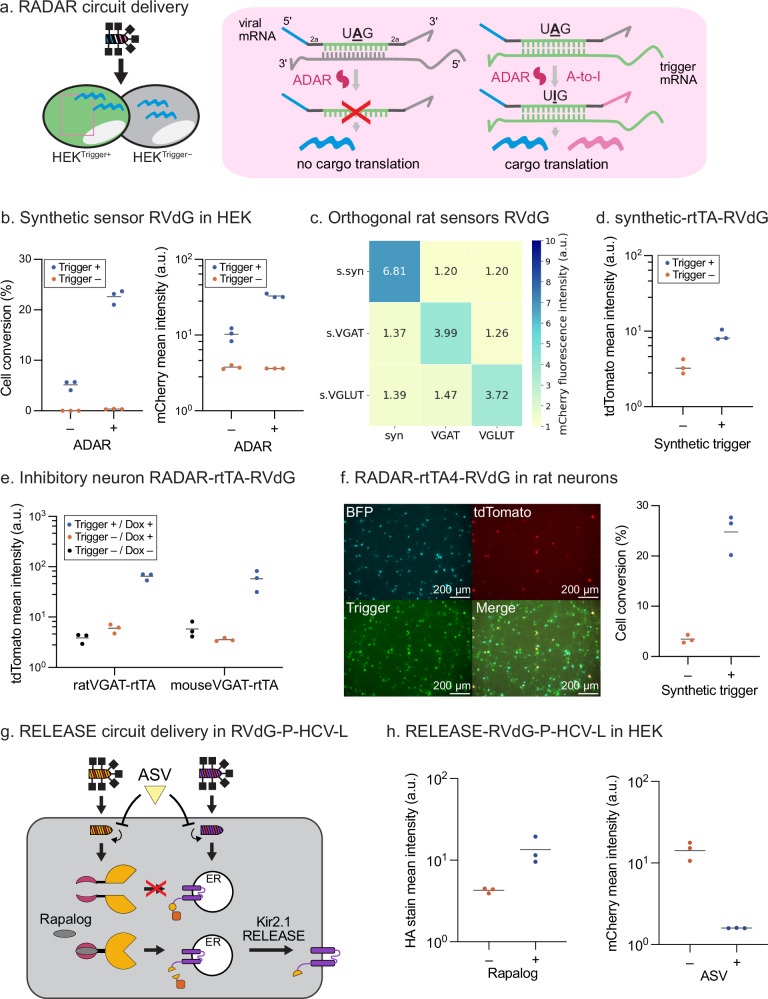
Two circuits control viral transgene specificity at transcript and protein levels. **a** Mechanism of ADAR-based RNA sensing. In the absence of a trigger mRNA, the stop codon prevents cargo translation. When the trigger mRNA is present, ADAR edits the sensor UAG to UIG (read as UGG), enabling translation of the downstream cargo. **b** Synthetic sensor activity in HEK cells. Left: flow cytometry quantification of sensor editing with or without ADAR. Cell conversion is defined as the percentage of cells expressing rabies mTagBFP co-transduction marker that also express cargo mCherry. Right: mCherry reporter mean fluorescence intensity increases in the presence of cognate trigger. **c** Orthogonal RADAR sensors for synthetic, rat Vgat, and rat Vglut transcripts tested in HEK cells. The heatmap shows mCherry mean fluorescence intensity of RADAR-RVdG in the presence of different trigger RNAs. **d** Reporter TRE-tdTomato mean fluorescence intensity in HEK cells for RADAR-rtTA-RVdG carrying the synthetic sensor, with or without synthetic trigger in the presence of dox. **e** Reporter TRE-tdTomato mean fluorescence intensity in HEKs cells for rabies carrying rat and mouse Vgat RADAR sensors, with or without cognate trigger and doxycycline. **f** RADAR-RVdG-rtTA4 in rat cortical neurons. Co-delivery of AAV-trigger and AAV-TRE-tdTomato, followed by infection with RADAR-rtTA4-RVdG, enables trigger-dependent tdTomato expression. Representative fluorescence images are shown. Scale Bar: 200 µm. **g** Schematic of RELEASE-Kir2.1 circuit in cells after splitRapaTEVP-RVdG-P-HCVP-L and RELEASE-Kir2.1-RVdG-P-HCVP-L delivery. ASV addition abolishes mCherry mean fluorescence intensity and rapalog addition increases HA-tag surface staining mean fluorescence. In (**b**, **d**, **e**, **f**, and **h**), each dot represents one biological replicate and the horizontal lines indicate the mean of data in each group (*n* = 3). Arbitrary units: a.u.

Recent advances in live RNA sensing leverage the natural double-stranded RNA (dsRNA) editing response of adenosine deaminase acting on RNA (ADAR)^25–27^, a mechanism compatible with RNA vector delivery. To harness this mechanism, we engineered a rabies virus encoding a live RNA sensing ADAR circuit (RADAR) as its cargo (Fig. 7a, left). Following viral delivery, the RADAR transgene is transcribed from the negative strand rabies genome and consists of three components: a transduction marker, a sensor sequence, and a cargo. In cells with abundant transcripts of interest (hereafter referred to as trigger), ADAR editing of the trigger-sensor dsRNA enables downstream translation of the cargo (Fig. 7a, right), coupling transgene cargo expression to endogenous RNA levels.

To characterize RADAR-RVdG, we engineered it with a previously characterized RNA sensor sequence corresponding to a 90 nt synthetic RNA trigger^25^ (Supplementary Fig. 7a). We tested live RNA sensing of the trigger transcript in HEK293 cells with or without the overexpression of the p150 isoform of ADAR1 (Supplementary Fig. 6). In this assay, both the cell conversion percentage - defined as the fraction of cells co-transduced with the synthetic RADAR-RVdG and cognate trigger that also express the cargo - and the cargo level, measured as mean mCherry intensity of all rabies and trigger plasmid positive cells, were significantly higher in cells overexpressing ADAR1p150 and target RNA (Fig. 7b).

In addition to synthetic RADAR-RVdG, we tested viruses carrying a previously characterized RADAR sensor for rat Vgat^26^ and a new RADAR sensor for rat Vglut1 (Supplementary Fig. 7b). As expected, the three RADAR-RVdG sensors were orthogonal and showed higher cell conversion and cargo level in response to cognate trigger RNA (Fig. 7c).

We then leveraged the modular design of RADAR to output diverse cargos, including Cre recombinase and rtTA transcription factor, as potential signal amplifiers. Using the synthetic sensor sequence, we engineered RADAR-RVdG virus with a Cre output (RADAR-Cre-RVdG) and infected HEK293 cells that carried the reporter plasmid DIO-mCherry. Although RADAR-Cre-RVdG exhibited elevated output in response to the synthetic trigger, we observed some leaky cargo expression at baseline compared to the previous fluorescent protein cargo design (Supplementary Fig. 7c and 7f). To introduce an additional layer of regulation, we incorporated the Tet-ON system by encoding rtTA4 as the cargo, enabling external control through doxycycline induction. Specifically, we engineered RADAR-RVdG with a rtTA4 output (RADAR-rtTA4-RVdG) and infected cells that carried the TRE-tdTomato reporter, which is selectively activated in the presence of both rtTA and doxycycline. We validated that cargo output was dependent on the presence of trigger RNA and dox using this RADAR-rtTA4-RVdG system with two previously characterized RADAR sensors for rat and mouse Vgat^24^ (Fig. 7e).

We again evaluated the engineered RADAR-rtTA4-RVdG in primary rat cortical neurons. Neurons were first transduced with two adeno-associated viruses (AAVs): one encoding TRE-tdTomato and the other delivering the synthetic trigger sequence (Supplementary Fig. 7e). A week later, neurons were infected with synthetic RADAR-rtTA4-RVdG and culture media was exchanged with media containing doxycycline. Neurons expressing the trigger AAV exhibited increased cell conversion and enhanced tdTomato fluorescence compared to those lacking the trigger (Fig. 7f).

Another compelling rationale for encoding specificity at the cargo level is the opportunity to implement protein circuits that engage with endogenous pathways such as protein trafficking. Notably, recent advances in circuit engineering have demonstrated the ability to regulate membrane protein trafficking in response to protease activity^28,29^. Building on this, we engineered RVdG-P-HCVP-L to incorporate a RELEASE circuit that retains HA-tagged Kir2.1 potassium channels in the endoplasmic reticulum (ER) and enables their surface trafficking in response to TEV protease activity. In addition, we engineered RVdG-P-HCVP-L with a rapalog-inducible split TEV protease to achieve external induction of the RELEASE circuit (Fig. 7g). In this final case, we examined the combination of viral life cycle control and such post-translational cargo control. When HEK cells were co-infected with RELEASE-Kir2.1-RVdG-P-HCVP-L and splitRapaTEVP-RVdG-P-HCVP-L, cell surface HA staining of Kir2.1 was elevated in cells treated with rapalog compared to untreated controls (Fig. 7h). Addition of ASV to the media reduced expression of the mCherry rabies co-transduction marker (Fig. 7h), demonstrating that protein-level circuit control can be combined with delivery control through the use of orthogonal proteases.

## Discussion

Because they remain outside the nucleus and thereby avoid the potential risk for insertional mutagenesis, RNA viruses are attractive candidates for synthetic circuit delivery vectors. In fact, while DNA vectors currently remain more prevalent in therapeutic applications, RNA viruses have received growing attention^48^, particularly in cancer therapy^49–51^ and in vaccine development^52,53^. Here we sought to address key challenges in making RNA viruses into a more engineerable, and safer, alternative to DNA vectors. Rabies virus, with its extensive history of engineering^54^ and applications in neuroscience^20,21^ provides a compelling model system. Importantly, all rabies viral vectors used in this study were glycoprotein-deleted (ΔG), rendering them incapable of spreading beyond initially transduced cells without complementation. As a step towards increasing the engineerability of rabies virus, this work had the dual purposes of integrating well-known mechanisms, such as pseudotyping into a single platform, while also designing new mechanisms to address outstanding challenges, such as escaper-resistant drug control of replication. We can now achieve multiple levels of specificity and control in rabies virus (Fig. 1). These levels include controlling viral secretion from sender cells (Figs. 2, 3c); achieving selective infection of target cells based on surface proteins, with external control through bispecific bridge proteins (Fig. 3); implementing conditional replication in target cells based on an intracellular protein (Fig. 4); designing escaper-resistant control of viral replication with the drug ASV (Figs. 5, 6); and modulating viral payload with post-transcriptional and post-translational circuits (Fig. 7). While each module will require further in-depth characterization, this framework establishes a foundation for future studies aimed at delivering programmable RNA vectors.

As a tool for basic research in neurobiology, the engineered rabies vectors introduced here could be immediately applicable for tracing synaptic connections requiring better control. Our results also highlight areas for improvement such as increasing production of EnvA pseudotyped virus and decreasing baseline signals. Upon further optimization, these vectors might help realize the powerful biomedical promise of synthetic biology. They could be delivered in sender cells that naturally home to a disease tissue^55^, exit sender cells only in the correct microenvironment, selectively enter target cells that display certain surface markers, replicate specifically in cells positive for specific markers, deliver genetic cargo that senses cell state and produces a therapeutic output, and finally be eliminated with a small-molecule drug. The self-replication of such vectors guarantees that their cargo will be highly expressed so as to effectively perform their functions, and the RNA nature of their genome and the ability to eliminate the virus from infected cells minimize the risk of a synthetic circuit leaving scars in the host genome or intracellular signaling pathways. While many challenges to this vision undoubtedly remain, the results here provide proof of principle demonstration of many of its core required capabilities.

The modules analyzed here are not specific to rabies virus and are likely transferable to other RNA viral vectors, including oncolytic RNA viruses. This framework could support the engineering of oncolytic platforms such as Vesicular Stomatitis Virus (VSV), which shares a similar negative-strand RNA genome, to further enhance immunostimulatory capacity and tumor selectivity^56,57^. For example, a RAS-sensing RELEASE circuit delivered by an oncolytic virus could conditionally secrete cytokines or display co-stimulatory ligands in cancer cells with elevated Ras activity, providing precision immunomodulation in tumor microenvironments^4,28^. Beyond oncolytic applications, these design principles can also be extended to alphavirus-derived RNA replicons, such as Venezuelan Equine Encephalitis Virus (VEEV), which are generated by replacing structural genes downstream of a sub-genomic promoter with a gene of interest to yield self-amplifying RNAs^58^. Prior efforts to tune expression from replicons have focused on exploring alternative viral RNA backbones^53^ or modifying sub-genomic promoter strength to adjust the magnitude and duration of transgene expression^59^. Our platform offers complementary strategies - for example, regulating nonstructural gene expressions required for replication with the small molecule-inducible HCVP module, or restricting sub-genomic translation of the transgene to specific cell types by placing a RADAR sensor upstream to the cargo. Such modules could broaden the design space for self-amplifying RNAs by introducing additional layers of temporal and cell-state specificity.

In addition, to realize the potential of RNA viral vectors, we will still need to address several challenges. First, excessive vector replication and cargo expression after delivery could generate toxicity by competing for host cellular resources, leading to detrimental effects on the patient. To address this, synthetic negative feedback circuits could be added to maintain vector and cargo levels below a tolerance threshold. Second, although rabies virus has evolved to counter detection and clearance by the immune system, it, like most other RNA viruses, is nevertheless immunogenic^60^. One solution would be to encode additional immunomodulating molecules to transiently suppress innate immunity while the vector and its cargo carry out their function. Third, although rabies vectors with inserts up to 3.7 kb long have been successfully produced and used for in vivo tracing^61^, it will be critical to systematically determine how delivery efficiency varies with the size and sequence identity of genetic cargo. If size limits prove to be inadequate for common applications, potential solutions include leveraging related RNA viruses with larger genomic capacity or distributing a circuit across interdependent vectors, each of whose replication depends on the other, to help maintain functional stoichiometric expression.

Future optimization of RNA viral vectors would benefit from novel methods to regulate RNAs and proteins, including the use of riboswitches^62^ and RNA-targeted nucleases^63–65^. The ability to bypass transcriptional control and host genome integration means that synthetic circuits made of RNAs and proteins are uniquely suitable cargo for RNA vectors, especially protein circuits that can be compactly encoded on a single transcript^7^. We anticipate that engineering rabies viral vectors and their cargo could drive the development of other homologous engineered RNA viruses, each adapted to specific therapeutic applications.

## Methods

### Plasmid construction

All constructs were generated using standard procedures including InFusion of linearized plasmids and PCR fragments and restriction–ligation of linearized fragments and annealed phosphorylated oligonucleotides. Inserts were generated from PCR, gBlock synthesis (IDT), or Twist Biosciences. A list of all plasmids reported in this manuscript is included in Supplementary Table 1. Plasmids and plasmid maps are available upon request from the corresponding author.

### Tissue culture

Flp-In™ T-REx™ 293 Cell Line (Human Embryonic Kidney cells that contain a single stably integrated FRT site at a transcriptionally active genomic locus, and stably expressing the tetracycline repressor protein) were purchased from Thermo Fisher Scientific (R78007). B7GG cell line (Baby Hamster Kidney cells that contain a stably integrated T7 RNA polymerase and rabies glycoprotein) and HEK-TVA cell line (Human Embryonic Kidney cells that contain stably integrated TVA receptor) were kindly gifted from Dr. Lindsay Schwarz at St. Jude Children’s Research Hospital. Gibco™ primary rat cortical neurons, Sprague Dawley, were purchased from Thermo Fisher Scientific (cat no. A36511).

HEK293 derived cells were cultured in media containing DMEM (Thermo Fisher Scientific, cat no. 11960-069) supplemented with 10% FBS (VWR, 76308-946), 1 mM sodium pyruvate (Thermo Fisher Scientific, cat no. 11360-070), 1 unit/ml penicillin, 1 μg/ml streptomycin, 2 mM L-glutamine (Thermo Fisher Scientific, cat no. 10378-016) and 1X MEM non-essential amino acids (Thermo Fisher Scientific, cat no. 11140-050) (293 media). B7GG-derived cells producing rabies virus were cultured in media containing DMEM supplemented with 2.5-5% FBS, 1 mM sodium pyruvate, 1 unit/ml penicillin, 1 μg/ml streptomycin, 2 mM L-glutamine, and 1X MEM non-essential amino acids (BHK media). Neurons were cultured in Neurobasal™ plus media (Thermo Fisher Scientific, cat no. A3582901) with 1X B-27™ plus supplement (Thermo Fisher Scientific, cat no. A3582801) and 1X GlutaMAX™ supplement (Thermo Fisher Scientific, cat no. A35050061) according to the Thermo Fisher Scientific manual on poly-D-lysine (Thermo Fisher Scientific, cat no. A3890401) coated plates. All cells were cultured in a humidity-controlled incubator under standard culture conditions (37 °C with 5% CO_2_).

100 ng/mL doxycycline (dox) or 4-epi-Tc was added whenever expression is needed from a CMV/TO promoter. Titration experiments were done with 4-epi-Tc (4-epitetracycline) to allow for graded induction of CMV/TO promoter. Trimethoprim (TMP) was delivered at 1 µM. Asunaprevinir (ASV) was delivered at 3 µM. Rapalog AP21967 (also known as A/C heterodimerizer, purchased from Takara Biosciences; catalog# 635056) is a synthetic rapamycin analog that can bind with FRB harboring the T2098L mutation. Neurons were treated to final concentrations of 200 nM rapalog, 3 µM ASV, or 1 µg/mL dox.

### Cell line construction

To generate cell lines with stably integrated transgenes antibiotic selection was performed. Flp-In™ T-REx™ 293 Cell Line or BHK21 cells were transfected in 24-well plates and transferred two days later into a 6-well plate containing selection media (Supplementary Table 2). After PiggyBac-based integration, monoclonal cell populations were established through limiting dilution, and preliminary screening was performed to identify clones with highest transgene expression using flow cytometry (Supplementary Table 2). Transgenesis using pOG44 Flp-recombinase into Flp-In™ T-REx™ 293 Cell Line resulted in singly-integrated cell lines. Cell lines will be available upon request.

### Production of packaging cell lines

B7GG was integrated with a transgene that encodes for constitutive co-expression of TEVP and Cerulean (B7GG-TCer). This packaging line was generated to permit efficient production of modified rabies genomes containing an essential protein tagged with a destabilizing domain, in which a TEVP cleavage site was inserted between the essential protein and the degron. TEVP cleavage of the degron stabilizes the essential protein allowing viral replication.

### Production of rabies viruses

Viruses were initially established using a protocol previously described and scaled for 24-well plates^66^. RVdG-P-DHFR and RVdG-P-GBP were produced in B7GG-TCer producer lines while RVdG, RVdG-P-HCVP-L, RADAR-RVdG, and RELEASE-RVdG viruses were produced in B7GG (Supplementary Table 2). Briefly, B7GG and B7GG-TCer cells were plated in 24-well plates at 0.05×10^6^ cells per well in 293 media. Cells were transfected with a DNA mixture containing 240 ng rabies genome, 120 ng pcDNA-SADB19N, 60 ng pcDNA-SADB19P, 60 ng pcDNA-SADB19L and 40 ng pcDNA-SADB19G. Media was changed one day after transfection to BHK media. Cells were transferred from a 24-well plate to a 6-well plate three days after transfection and maintained in BHK media. Fresh BHK media was added every day to facilitate viral spread. Viral spread was checked every day using fluorescent microscopy. Supernatant collection only proceeded when the virus had visibly infected the majority of the population.

Amplified rabies viruses were concentrated and purified as previously described to generate starter viral stocks^66^. Briefly, infected producer lines were expanded to three 15 cm dishes and exchanged with fresh BHK media the following day. After three days, conditioned media was collected and filtered through at 0.45 um filter. The supernatant was concentrated in an ultracentrifuge at 70,000 × g for 2 hr at 4 °C on a 20% sucrose pad or using MilliporeSigma™ Amicon™ Ultra-15 centrifugal filter units (cat no. UFC901008) in a centrifuge at 4000 × *g* for 45 minutes and then again with 1X Hank’s Balanced Salt Solution (HBSS). Viral pellets were resuspended in HBSS and stored at −80 °C for future use. Pseudotyped EnvA rabies virus was purchased from Janelia Farms or produced using EnvA-BHK cells previously described^66^.

### Production of bridge protein cell lines

#### In vivo production of bridge proteins

The extracellular domain of ASLV-A envelope protein from the plasmid pAAV-TRE-HTG (Addgene) and the targeting domain encoding the Gbp6 nanobody^39^ were combined by PCR and cloned into a PiggyBac transfer vector under a synthetic constitutive promoter (Supplementary Table 1). Flp-In™ T-REx™ 293 cells were stably integrated to create a polyclonal bridge protein secretion line. To collect produced bridge proteins, the stable line was seeded at a density of 0.1 × 10^6^ cells per well of a 6-well plate and cultured under standard conditions overnight. The media was exchanged the following day for BHK21 media, and cells were cultured over two days with additional fresh media applied the second day. Conditioned media was collected and centrifuged at 4000 × g for 20 minutes to remove cellular debris. 300 uL of conditioned media was applied in each condition unless otherwise noted.

### In vitro production of bridge proteins

Bridge proteins were purified at the Caltech Protein Expression Center. Briefly, 6XHIS-tagged bridge proteins encoding plasmids (Supplementary Table 1) were transfected into Expi293FTM cells and conditioned media was harvested four days post-transfection. The supernatant was spun at 2000 × *g* for 10 minutes and proteins were purified with Ni-NTA affinity chromatography (Cytiva, 5 mL HisTrap). Expression and purification were checked by SDS-PAGE and Coomassie Blue staining. Protein concentration was quantified using PierceTM 660 nm (Thermo Fischer, cat no. 22662) assay.

### DNA transient transfection

293 cells were seeded at a density of 0.05 × 10^6^ cells per well of a 24-well plate and cultured under standard conditions overnight. The following day, the cells were transfected with plasmid constructs using Lipofectamine LTX (Thermo Fisher) as per manufacturer’s protocol. For RADAR-RVdG experiments in HEK, cells were plated for 30% confluency, infected with rabies 4 hours later, and then transiently transfected with plasmid constructs using Lipofectamine 3000 (Thermo Fisher) as per manufacturer’s protocol two days later. Each 25 well was transfected with 0.75 μl of Lipofectamine 3000 reagent.

### Adeno-associated virus (AAV) generation and transduction

The AAVs were manufactured through the Stanford Gene Vector and Virus Core (GVVC). Plasmids were grown up in 50 mL cultures of LB and then extracted using Qiagen plasmid midiprep kit, as per manufacturer’s instructions. GVVC produced a set of AAVs with the AAV8 (with Y733F mutation) or AAV-DJ serotypes. The AAVs were measured for their ITRs and used to transduce rat cortical neurons at different multiplicities of infection (MOI) ranging from 25,000 to 100,000 in a total of 1 mL of neuron media per well on DIV3.

### Infection with rabies

Infected producer cell lines B7GG and B7GGTCer were plated to 60% confluency in 6-well plates. The following day, the media was changed to 2 mL of fresh BHK media. Two days later an additional 1 mL of BHK media was added. Virus containing media was collected on the third day and supernatant was spun down for 5 minutes at 4000 × *g* to remove cellular debris. Supernatant from these freshly prepared stocks were used directly in RVdG-P-GBP (Fig. 3) and RVdG-P-HCVP-L (Figs. 4, 5, Supplementary Figs. 3, 4) experiments. Viral amount was determined by volume, as indicated on figures. Infection using purified RVdG-EnvA was performed by calculating MOI based on the reported viral titer provided by Janelia Farms. For experiments containing sender cells (Figs. 1, 2c), sender cells were plated at 30% confluency and the following day media was exchanged with 1 mL BHK media and 1 mL of freshly prepared RVdG supernatant, as described above.

All experiments were conducted in 24-well plates. In co-culture experiments, 30,000–45,000 cells for each target population were counted and pre-mixed prior to plating. Specifically, for the co-culture performed in Fig. 3b, the expression of Citrine was induced with doxycycline overnight prior to plating and was present throughout the experiment (Supplementary Table 2, refer to HEK^doxCit^). For single-target cell population experiments, the cells were seeded at 75,000 cells per well. Infected sender cells, virus-containing supernatant, purified virus, bridge protein, and compounds were added to each well immediately after target cells were plated unless otherwise noted.

For RADAR-RVdG experiments in HEK cells, rabies virus was buffer-exchanged and concentrated using Amicon filters, then serially diluted to identify an MOI of 0.5-1. These volumes were subsequently used for the experiments shown in Fig. 6b–d. Neurons were infected with RADAR-RVdG 7 days after AAV transduction on DIV3, whereas infections with RVdG-P-HCVP-L, RELEASE-RVdG, and splitRapaTEVP-RVdG were performed on DIV7 at an MOI of 1–10 (based on serial dilutions in neurons).

### Flow cytometry and data analysis

Flow cytometry samples were performed in biological triplicates. Three days after transfection or infection, cells were prepared for flow cytometry by trypsinizing with 40–100 μL of 0.05% trypsin for 1-5 min at room temperature. Protease activity was neutralized by resuspending the cells in buffer containing 100 μL of HBSS with 2.5 mg/ml Bovine Serum Albumin (BSA) and 2 mM EDTA (Thermo Fischer Scientific, cat no. 15575020). Cells were then filtered through 40 μm Falcon TM Cell Strainers (Thermo Fischer Scientific, cat no. 08-771-1) and analyzed by flow cytometry (MACSQuant VYB, Miltenyi, CytoFLEX, Beckman Coulter, or Biorad ZE5 Cell Analyzer). We used the EasyFlow Matlab-based software package developed in-house by Yaron Antebi to process flow cytometry data.

### Fluorescent signal quantification from flow cytometric measurements

Figures 1–4, 6 and Supplementary Fig. 1 and 7 present quantitative analysis of flow cytometry measurements. For each sample in a comparison group (experiments performed in the same batch and data shown on the same plot), we reported the percent infected as identified by mCherry+ cells. Viable cell populations were determined as events that registered above 10^5^ in both the FSC and SSC channels. A manual gate was drawn around a single contiguous population that excluded both cellular debris and diagonal streaking events.

### Co-culture gating

The fluorescence values of each cell line were determined in the constitutive fluorescent signal channel (Citrine or IFP in most cases). Each cell line was analyzed independently. For example, for a co-culture containing HEK^wt^ and HEK^Cit^, monoculture samples of HEK^wt^ and HEK^Cit^ were each analyzed independently in the Citrine fluorescent channel. For analysis, we fit the fluorescence log distribution with skew Gaussian distributions, i.e. *n* × normcdf(x,m,k) × normpdf(x,m,s) in Matlab using non-linear least-square fitting, and reported the mode of the resulting fit. Here, the normcdf (x, m, k) and normpdf (x, m, s) functions are cumulative probability density and probability density functions for Gaussian distributions, respectively. The parameter n is a normalization factor, the parameter m is the mean of each Gaussian distribution, s is the standard deviation of the probability density function that parameterizes the width of the distribution, and k is the standard deviation of the cumulative probability density function that parameterizes the skewness of the distribution.

### Percent infected calculation

The mode of the resulting fit is used to gate different cell types from the co-cultured samples. For monoculture experiments, gating was not performed. For all cells within the gate in each sample, we fit a cubic spline and identified the local minima between the two peaks of mCherry+ and mCherry- cells. An average local minimum was calculated across all bimodal samples. We use this average local minima as a threshold between non-infected/weakly infected cells (mCherry-) and highly infected cells (mCherry + ). A percent infected metric is calculated from the resulting histogram by calculating the area both above and below the threshold:

$${{\rm{Percent}}}\; {{\rm{infected}}}=({{\rm{Area}}}\; {{\rm{above}}}\; {{\rm{threshold}}})/({{\rm{Total}}}\; {{\rm{area}}})\times 100$$

### RADAR-RVdG gating

Cells were gated based on expression of the mTagBFP co-transduction marker (mTagBFP+) and the GFP trigger plasmid marker (either cognate or non-cognate). Gating thresholds for positive and negative populations were established using autofluorescence signals from untransduced control wells lacking fluorescent markers. For cargo reporters (mCherry or tdTomato), positive cell gating was determined by comparing fluorescence histograms of untransfected negative control wells with those transiently transfected with reporter plasmids and positive control plasmids (Cre or rtTA4).

### Cell surface staining

For experiments measuring the surface display of Kir2.1, cells were fixed in 4% PFA (Thermo 16% PFA cat no. 28908, 4% in DPBS) and stained as previously described^26^. Cells were transferred to U-bottom 96-well plates and pelleted by centrifugation at 500 × *g* for 5 mins. Cells were then incubated with 100 μl of corresponding antibody dilutions in flow buffer for 30 minutes at room temperature. Cells were incubated in 1:500 dilution of anti-hemagglutinin antibody (HA, Abcam; catalog# ab137838), followed by incubation with 1:1000 dilution of a donkey anti-rabbit IgG conjugated to Alexa-647 (Abcam, cat no. ab150075). After staining, cells were washed twice with flow buffer and then strained using a 40 μm cell strainer. Cells were analyzed by flow cytometry (BioRad ZE5 Cell Analyzer) as previously described.

### Sequencing virus genomes P-DHFR and P-GBP

Three independent viral cultures were passaged for 2-4 months prior to genome sequencing. Reverse transcription of viral RNA was performed on samples using the Thermo Fisher Scientific protocol for SuperScript IV. PCR amplification of the genome using forward primer (5’-ACCCTCCAGGAAAGTCTTC-3’) and reverse primer (5’-AATAGGGTCATCATAGACCTCTC-3’) were gel purified and submitted for Sanger sequencing with Laragen Sequencing.

### Time-lapse microscopy

For time-lapse imaging of rabies dilution (Fig. 5, Supplementary Fig. 3, Supplementary Fig. 4, Supplementary Movies 1 and 2) 5000 H2B-Citrine were plated on 24-well glass- bottom plates (CellVis). Cells were induced with 100 ng/mL doxycyline overnight in normal culturing conditions. The following morning, the media was replaced with imaging media containing FluoroBrite DMEM (Thermo Fisher) supplemented with 10% FBS, 1 mM sodium pyruvate, 1 unit/ml penicillin, 1 μg/ml streptomycin, 2 mM L-glutamine, and 1X MEM non-essential amino acids and 100 ng/mL doxycycline. All time-lapse images were acquired on an inverted Olympus IX81 fluorescence microscope with Zero Drift Control (ZDC), an ASI 2000XY automated stage, iKon-M CCD camera (Andor, Belfast, NIR), and a 20X PAN-FL objective (1.42 NA). Fluorophores were excited with an X-Cite XLED1 light source (Lumen Dynamics). Cells were kept in an environmental stage-top chamber enclosing the microscope, with humidified 5% CO 2 flowing at 37 °C (Okolab H301-K stage top with an O 2 -CO 2 UNIT-BL mixer). Microscope and image acquisition were controlled by Metamorph software version 7.10 (Molecular Devices).

Imaging started approximately 2 hours after changing the media to fluorescent imaging media. B7GG lines producing virus were cultured for three days in imaging media. Conditioned media were collected and centrifuged at 4000 × *g* to pellet cellular debris. 250 uL of the centrifuged supernatant containing virus was added after approximately 2 hours of imaging to infect cells. The initial infection was established for 24 hours. To observe the removal of the rabies genome with prolonged ASV treatment, cells were washed with imaging media three times to remove residual virus and then incubated with imaging media containing 3 μ M of ASV. The media was changed every 24 hours. To confirm that the virus does not re-emerge after ASV removal, the ASV-containing imaging media were replaced with normal imaging media after 7 days. Images were acquired every 90 minutes throughout the duration of the movie. Cells that were in the field of view before infection and remained alive and visible in the field of view without death for at least five days were used for initial data analysis through manual inspection.

In the preliminary experiment (Supplementary Fig. 4, Supplementary Movie 2), cells were infected for 17 hours and media was changed every 3 days over 6 days. Images were acquired every 60 minutes. For analysis, a similar selection criteria was performed in which cells that were infected and remained within the field of view for five days were used for data analysis. Additionally, we excluded from analysis fields of view that were out of focus, near the edge of the well, or exhibited clumping or apoptosis within the last day of imaging.

### Neuron imaging and analysis

On the day of imaging, neuronal media were replaced with supplemented Neurobasal Medium without phenol red (Thermo Fisher Scientific, cat. No. 12349015), with ~75% of the total well volume exchanged. Imaging was performed on an EVOS M7000 using DAPI (AMEP4950), GFP (AMEP4951), RFP (AMEP4952), and Cy5.5 (AMEP4973) filter cubes, with five fields captured per well. Images were analyzed using ImageK (Figi for MacOS) by first subtracting the background for all channels (process→subtract background) using a 200-pixel window (rolling ball setting in ImageJ). Binary masks were generated using thresholding and binarization, with thresholds determined from negative control neurons lacking fluorescent markers. Data for Fig. 4e were manually counted, while data for Fig. 6 and Supplementary Fig. 7 were quantified using a custom Fiji macro script (available for download).

### Movie analysis

#### Data processing

mCherry mean intensity values were calculated based on total levels of fluorescence in the mCherry fluorescent channel as identified by the position of cells in the Citrine fluorescent channel. To systematically identify the position of cells, the total constitutive Citrine signal was used to segment each image. The resulting segmentation mask was used to calculate the number of mCherry+ pixels within each region. To capture the magnitude of rabies expression within infected cells, the mean intensity was calculated for each mCherry+ region and averaged for each time point. The fraction infected metric was calculated by identifying the mCherry+ areas within the Citrine+ areas.

We first estimated the fraction of cells infected by the rabies virus. We used the constitutive Citrine signal to generate a mask for all cells, where a pixel is considered to belong to a cell if its Citrine intensity is > 400, as determined by an Otsu threshold of the first Citrine-containing image. Within the Citrine mask, pixels are considered rabies-positive when mCherry intensity is greater than a moving Otsu threshold to account for fluctuations in background signal. As such rabies-positive area divided by the area of the Citrine mask (mCherry area norm. in Fig. 5, Supplementary Fig. 3 and 4) is reported as a proxy for the fraction of rabies-infected cells in each image. The constitutive Citrine signal is localized to the nucleus and may underestimate the rabies-positive area, especially towards the beginning of the imaging when rabies-encoded mCherry forms puncta in the cytoplasm. In addition to thresholding the mCherry signal, we also estimated the quantitative level of rabies infection by calculating the total mCherry intensity within each Citrine-positive region of each image and averaging across these regions (mCherry mean int. in Fig. 5, Supplementary Fig. 3 and Supplementary Fig. 4) as a proxy for the level of rabies infection in that image.

### Movie generation

For visualization purposes, the time point at which maximum mCherry intensity occurs for each movie was determined and used to rescale all images between the 5^th^ and 99.5th percentile intensity values.

### Biosafety

All experiments involving recombinant viral vectors were approved by the Institutional Biosafety Committee at Stanford University and conducted in Biosafety Level 2 (BSL-2) laboratories in accordance with institutional biosafety guidelines.

### Statistical analysis

Values are reported as the means from at least three biological replicates, representative of two independent biological experiments. The movies are technical replicates, done in parallel. Neuron experiments were done in biological triplicate. For experiments comparing two groups, a Bonferroni-corrected two-tailed Student’s t-test was used to assess significance. Data was considered statistically significant when the p-value was less than 0.05. All statistical analysis was performed using Prism 10.0 (GraphPad).

### Reporting summary

Further information on research design is available in the Nature Portfolio Reporting Summary linked to this article.

## Supplementary information

Supplementary Information

Description of Additional Supplementary Files

Reporting Summary

Supplementary Movies 1 and 2

Transparent Peer Review file

## Data Availability

The datasets generated and analyzed used during the current study are available at 10.5281/zenodo.18945530 or from the corresponding author.

## Code availability

The computer codes used in the current study are available at 10.5281/zenodo.18945530. Flow cytometry data analysis software used for this study is available at https://antebilab.github.io/easyflow/.
