# Supplementary material for: Engineering multiple levels of specificity in an RNA viral vector
Source: Nat Commun. 2026 Apr 2;17:4758. doi: 10.1038/s41467-026-71033-7 (PMC13216580; doi:10.1038/s41467-026-71033-7)
Supplement: Supplementary file 1 — Supplementary Information [file 41467_2026_71033_MOESM1_ESM.pdf]

SUPPLEMENTARY FIGURES

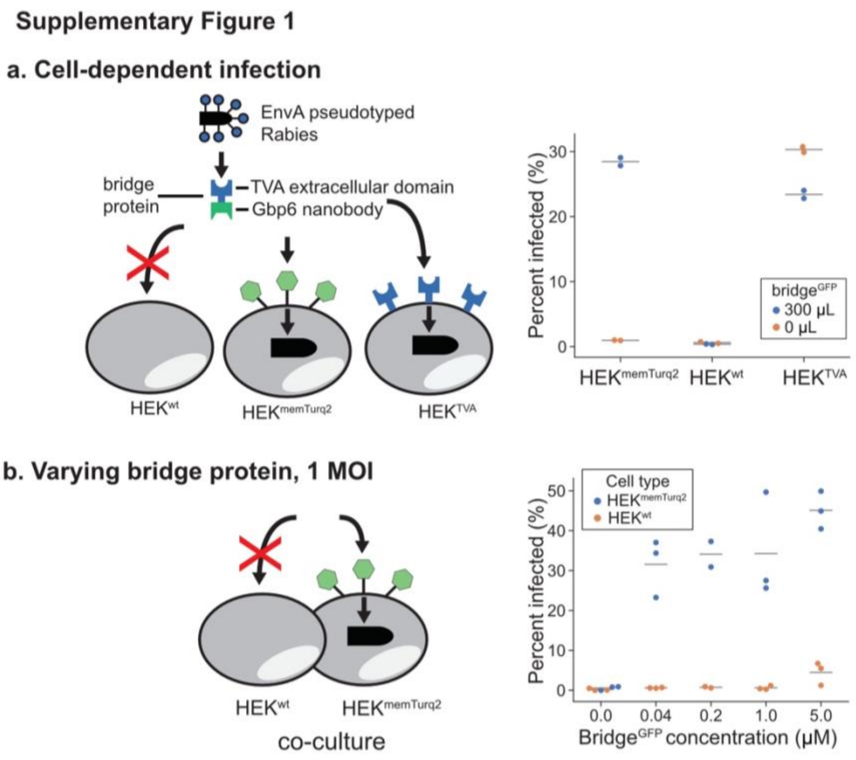

Supplementary Figure 1: Pseudotyped rabies viral entry is dependent on presence of bridge and cell-surface marker. **(a)** RVdG-EnvA virus infection of independently cultured $HEK^{memTurq2}$, HEK^{wt}, and HEK^{TVA} with or without $bridge^{GFP}$. **(b)** RVdG-EnvA and varied $bridge^{GFP}$ concentrations infection of a co-culture of $HEK^{memTurq2}$ and HEK^{wt}. Each dot represents one biological replicate and the horizontal lines indicate the mean of data in each group (n=2 for a, n=3 for b).

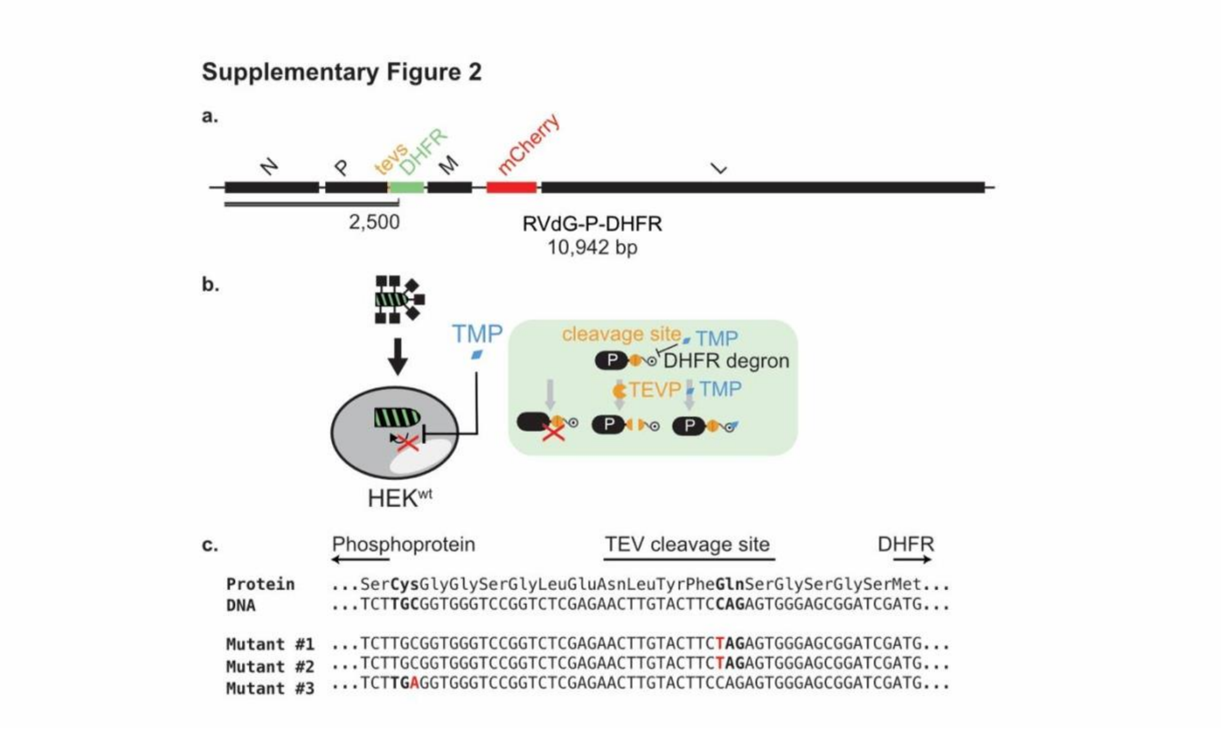

Supplementary Figure 2: C-terminal tagging of phosphoprotein exhibits evolutionary escape. (a) Recombinant rabies genome, RVdG-P-DHFR. **(b)** Schematic detailing phosphoprotein regulation through C-terminal DHFR degron tagging. The phosphoprotein is stabilized by removal of the DHFR degron either through TEVP cleavage (orange pac-man) of the corresponding cleavage site (orange circle)**.** Trimethroprim (TMP, blue diamond) inhibits the degron and stabilizes the reporter. **(c)** Sequencing of three escape mutants identified two distinct missense mutations. Coding sequences and corresponding amino acid sequences of Phosphoprotein, TEVP cleavage site, and DHFR are indicated above. Red indicates single nucleotide mutations and bold indicates mutated codons.

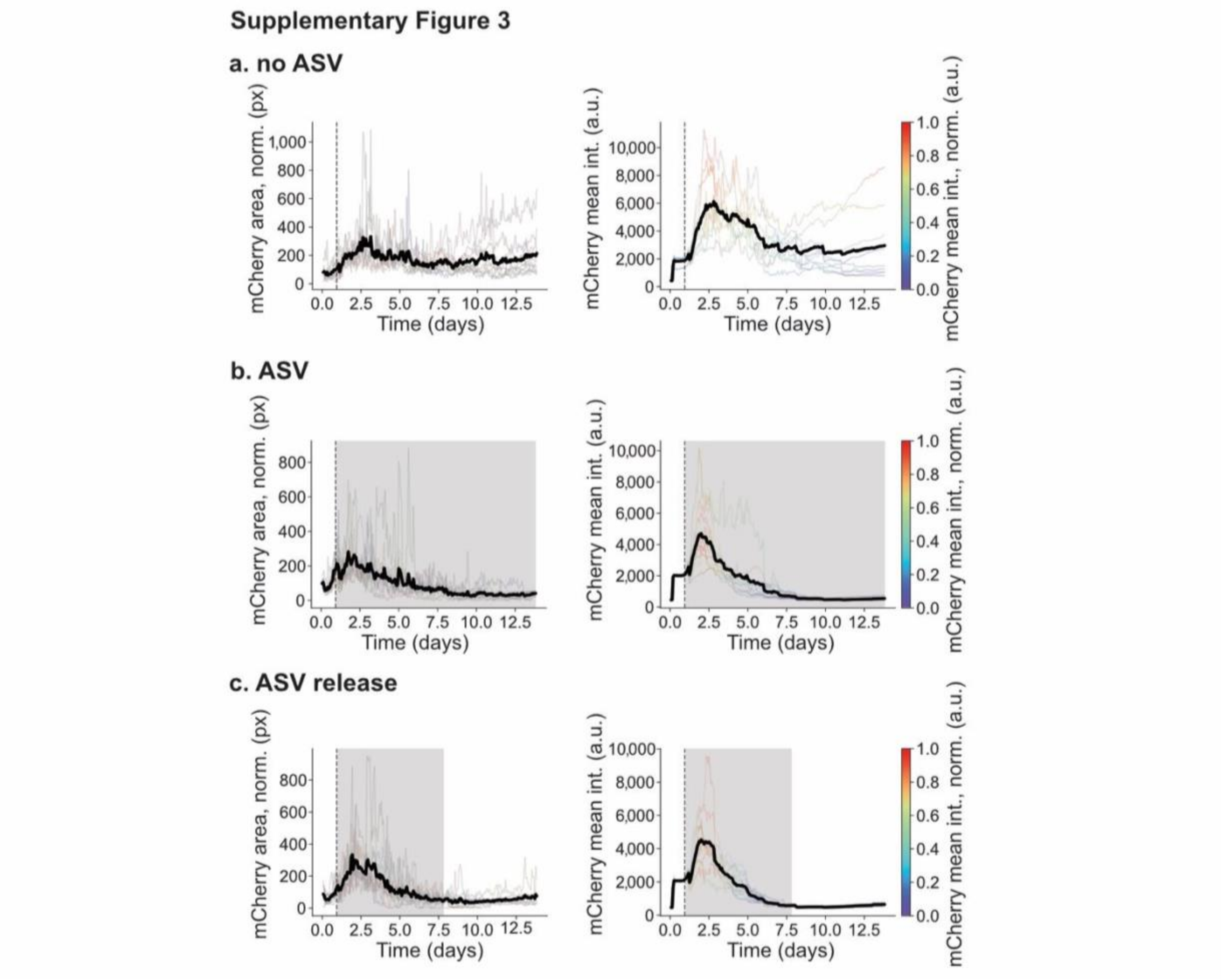

Supplementary Figure 3: Time-lapse microscopy shows viral removal of non-infected cells. Plots shown from Figure 5d with full y-axis. HEK target cells expressing H2B-Citrine were infected for 24 hours with RVdG-P-HCVP-L (dotted line) and then cultured in media containing no ASV **(b, no ASV**), continuous ASV **(c, ASV)**, or ASV for 6.9 days and then for 5 days in media containing no ASV **(d, ASV release)**. Grey shading indicates the presence of ASV in media. Transparent and black lines respectively represent traces from individual movies and the mean of those traces. Fraction of infected cells as indicated by “mCherry area, norm (px)” **(b, c, d, left column)** was calculated as the fraction of mCherry+ pixels within the H2B-Citrine+ mask. Individual traces for mCherry intensity are color-scaled from purple to red, where mCherry intensity values are normalized by the maximum mCherry intensity within each trace. The slight increases at late times in the mean mCherry intensity traces represent cellular autofluorescence.

Supplementary Figure 4

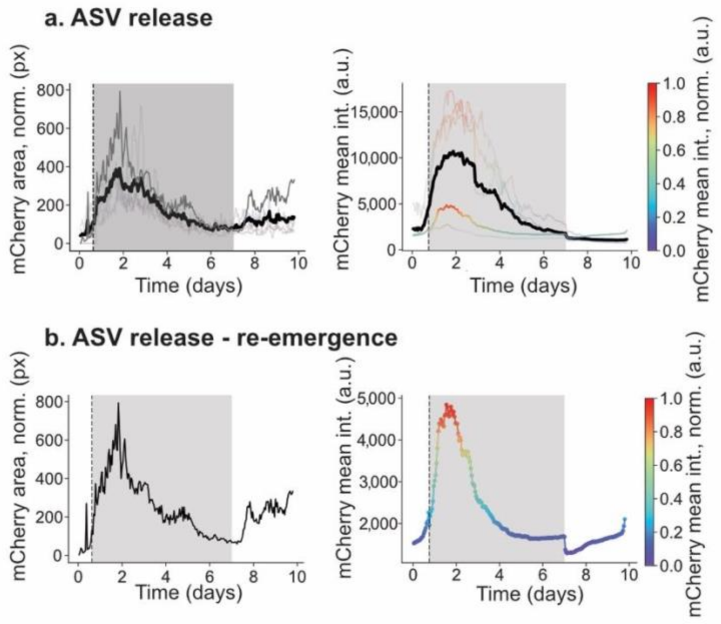

Supplementary Figure 4: Time-lapse microscopy shows viral reemergence in a preliminary experiment. (a) HEK target cells expressing H2B-Citrine were infected for one day with RVdG-P-HCVP-L (dotted line) and then cultured in media containing ASV for 6.2 days with media changed every three days and then for 3 days in media containing no ASV. Grey shading indicates the presence of ASV in media. Transparent and black lines respectively represent traces from individual movies and the mean of those traces. Opaque lines indicate viral re-emergence, shown independently in **(b)**.

Supplementary Figure 5

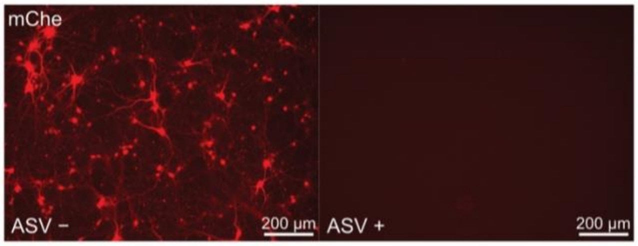

Supplementary Figure 5: Representative images of RVdG-P-HCVP-L infection in primary rat cortical neurons. DIV7 neurons were infected with RVdG-P-HCVP-L and maintained for 5 days with daily addition of 100-200 µl fresh media, either with or without ASV. Left: mCherry signal 5 days post-infection without ASV. Right: mCherry signal 5 days post-infection with ASV treatment. The two images are shown at the same contrast and brightness settings.

Supplementary Figure 6

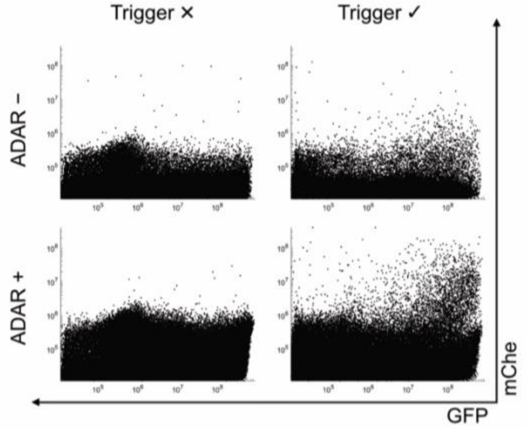

Supplementary Figure 6: Representative flow cytometry scatterplots of RADAR-RVdG activity. Trigger expression (GFP) and RADAR-RVdG cargo expression (mCherry) in HEK cells. Plots depict synthetic sensor-mCherry-RVdG cargo signal under four conditions: with or without ADARp150 overexpression, and with either cognate or non-cognate GFP-trigger plasmids.

Supplementary Figure 7

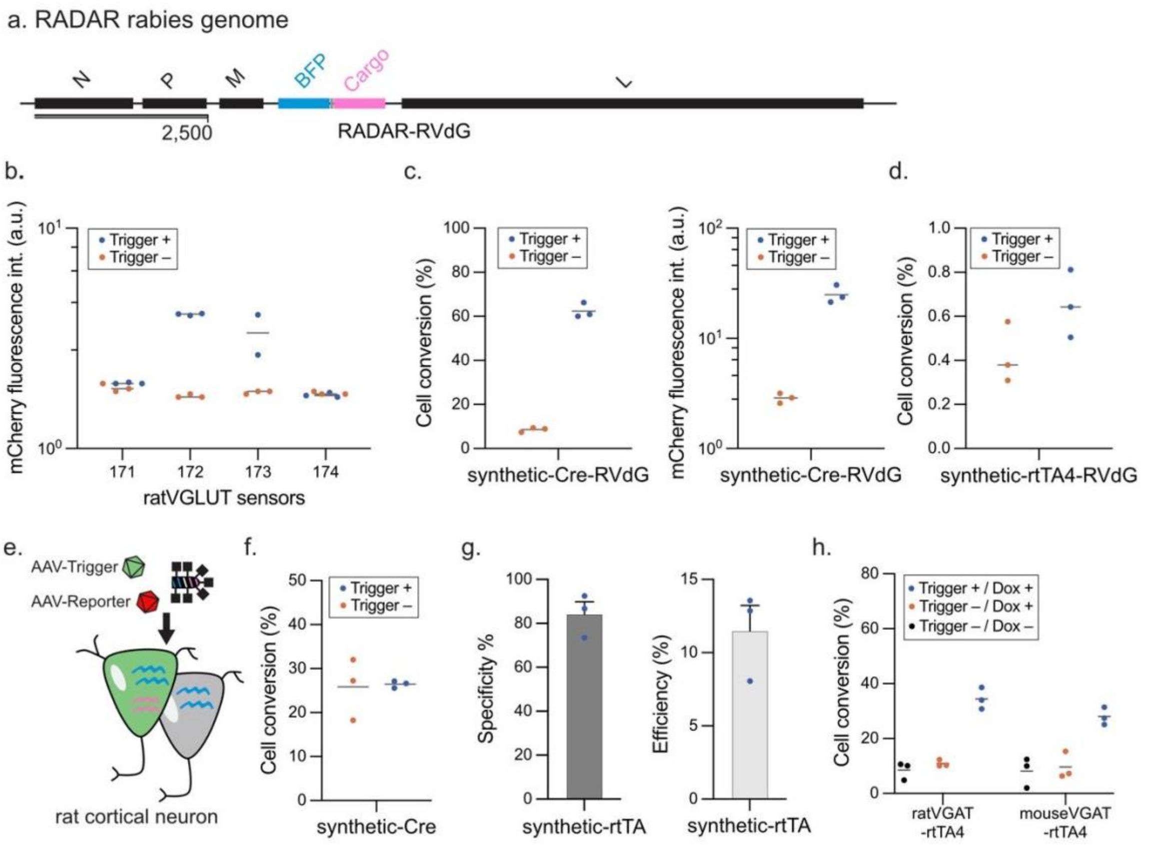

Supplementary Figure 7: Characterization of RADAR-RVdG components. (a) Schematic of RADAR-RVdG design including mTagBFP2 co-transduction marker, sensor sequence, and a cargo payload. **(b)** mCherry cargo signal for new RADAR sensor sequences targeting rat Vglut; sensor 172 was used for Figure 7c. **(c)** RADAR-synthetic-Cre-RVdG activity in HEK cells co-transfected with reporter DIO-mCherry plasmid. Left: Percentage of cells positive for rabies (mTagBFP2+) and cognate or non-cognate trigger plasmid (GFP+) that are also positive for cargo (mCherry+). Right: mCherry mean fluorescence intensity for the same transduced subpopulation of mTagBFP2+ and GFP+ cells. **(d)** Conversion percentage of HEK cells transfected with RADAR-synthetic-rtTA4-RVdG and trigger plasmids, corresponding to reporter tdTomato fluorescence mean intensity shown in Figure 7d. **(e)** Experimental schematic of primary rat cortical neurons transduced on DIV3 with AAV-GFP-trigger together with AAV-DIO-tdTomato or AAV-

TRE-tdTomato, followed by rabies infection 6-10 days later. **(f)** Neuron conversion percentages of AAV-GFP-trigger positive versus trigger negative neurons show similar outcomes even at lower MOIs of AAV or RADAR-synthetic-Cre-RVdG (data not shown). **(g)** The specificity and efficiency of RADAR-synthetic-rtTA4-RVdG infection in neurons from Fig. 7f. Specificity was defined as the percentage of tdTomato+ cells also positive for GFP-trigger. Efficiency was defined as the percentage of GFP-trigger+ and rabies mTagBFP2+ cells that were also reporter tdTomato+. **(h)** Cell conversion data corresponding to Fig. 7e for RADAR-rtTA4-RVdG targeting rat Vgat or mouse Vgat transcript. Each dot represents one biological replicate and the horizontal lines indicate the mean of data in each group (n=3 for b-d and f-h). Data in g are mean$\pm$s.e.m.

Supplementary Figure 8

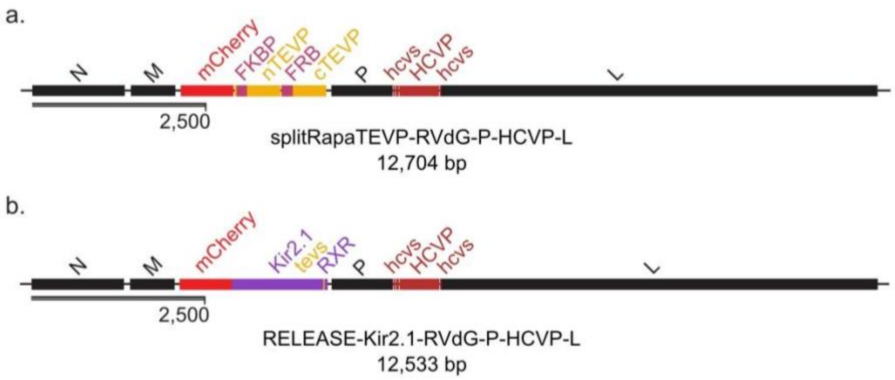

Supplementary Figure 8: Schematics of RELEASE-RVdG-P-HCVP-L components. (a) Split TEVP containing rapalog-binding domains (FKBP and FRB) was inserted into RVdG-P-HCVP-L genome. A T2A self-cleaving sequence separates mCherry and split TEVP. FKBP-nTEVP is anchored to the endoplasmic reticulum (ER) via a p450 domain to enhance TEV protease activity at the ER. **(b)** The Kir2.1 coding sequence, engineered with a TEV cut site and RXR retention motif at the C terminus, was fused downstream of mCherry in the RVdG-P-HCVP-L genome.

Supplementary Table 1	
**Construct**	**Intent**
PB-CMVTO-TEVP-Cer-Zeo	Cell line generation
CMV-TO-G-Cit (FLP-IN)	Cell line generation
CMV-TO-EnvA-IFP3XNLS (FLP-IN)	Cell line generation
PB-CMV-Cit	Cell line generation
PB-pEF1s-P-TEVP-IFP3XNLS-Neo	Cell line generation
PB-pEF1s-TVAecd-Gbp6HIS-Cer-Neo	Cell line generation
CMV-TO-memGFP (FLP-IN)	Cell line generation
PB-pEF1s-memTurqoise2-Neo	Cell line generation
PB-CMV-TO-Citrine-EF1a-Neo	Cell line generation
PB-pEF1s-Citrine (FLP-IN)	Cell line generation
CMV-TO+Cit-Int (FLP-IN)	Cell line generation
pSADdeltaG-mCherry-2A-L	Rabies genome
pSADdeltaG-mCherry-PteGBP-L	Rabies genome
pSADdeltaG-mCherry-PHCVL	Rabies genome
pSADdeltaG-mCherry-PteDHFR-L	Rabies genome
pSADdeltaG-mTagBFP2-synthetic-mCherry	Rabies genome
pSADdeltaG-mTagBFP2-synthetic-Cre	Rabies genome
pSADdeltaG-mTagBFP2-synthetic-rtTA4	Rabies genome
pSADdeltaG-mTagBFP2-ratVgat-mCherry	Rabies genome
pSADdeltaG-mTagBFP2-ratVgat-rtTA4	Rabies genome
pSADdeltaG-mTagBFP2-mouseVgat-mCherry	Rabies genome
pSADdeltaG-mTagBFP2-mouseVgat-rtTA4	Rabies genome
pSADdeltaG-mTagBFP2-ratVglut-mCherry	Rabies genome
pSADdeltaG-mTagBFP2-ratVglut-rtTA4	Rabies genome
pSADdeltaG-mChe-Kir2.1-tevs-RXR-PHCVL	Rabies genome
pSADdeltaG-mChe-splitRapaTEVP-PHCVL	Rabies genome
pAAV-EF1a-DIO-tdTomato-WPRE	reporter
pAAV-TRE3-tdTomato-WPRE	reporter
pAAV-Syn-GFP-stop-synthetic	synthetic trigger
pAAV-Syn-GFP	non-cognate trigger
SFFV-mTagBFP2-SGc(ratSLC17A7s1)cSG-mCherry (FLP-IN)	ratVglut RADAR sensor 1
SFFV-mTagBFP2-SGc(ratSLC17A7s2)cSG-mCherry (FLP-IN)	ratVglut RADAR sensor 2
SFFV-mTagBFP2-SGc(ratSLC17A7s3)cSG-mCherry (FLP-IN)	ratVglut RADAR sensor 3
SFFV-mTagBFP2-SGc(ratSLC17A7s4)cSG-mCherry (FLP-IN)	ratVglut RADAR sensor 4
CMV-TO-EGFP-stop-synthetic	synthetic trigger
CMV-TO-EGFP	GFP marker/negative control
CMV-TO-EGFP-stop-ratVgat(255)	rat Vgat trigger
CMV-TO-EGFP-stop-mouseVgat(full)	mouse Vgat trigger
CMV-TO-EGFP-stop-ratVglut(UTR)	rat Vglut trigger

Supplementary Table 1: List of constructs used to generate cell lines and rabies viruses.

Supplementary Table 2										
**Cell Line**	**Type**	**Generated From**	**Construct**	**Integration Method**	**Selection Process**	**Concentration (µg/mL)**	**Monoclonal or Polyclonal**	**Color**	**Final Resistances**	**Figure**
G-Cit	Sender	Flp-In™ T-REx™ 293	CMV-TO-G-Cit (FLP-IN)	pOG44	Hygromycin	50	Monoclonal	mCitrine	Hygromycin	2b
EnvA-IFP	Sender	Flp-In™ T-REx™ 293	CMV-TO-EnvA-IFP3XNLS (FLP-IN)	pOG44	Hygromycin	50	Polyclonal	IFP2	Hygromycin	3c
PTEVP	Sender	EnvA	PB-pEF1s-P-TEVP-IFP3XNLS-Neo	Piggybac Transposase	Geneticin	400	Monoclonal	IFP2	Hygromycin/Geneticin	
HEK-memGFP	Reciever	Flp-In™ T-REx™ 293	CMV-TO-memGFP (FLP-IN)	pOG44	Hygromycin	50	Polyclonal	mGFP	Hygromycin	3a
HEK-memTurq2	Reciever	Flp-In™ T-REx™ 293	PB-pEF1s-memTurqoise2-Neo	Piggybac Transposase	Geneticin	400	Monoclonal	mTurqoise2	Geneticin	3b, 3c, S1
HEK-doxCit	Reciever	Flp-In™ T-REx™ 293	PB-CMV-TO-Citrine-EF1a-Neo	Piggybac Transposase	Geneticin	400	Polyclonal	mCitrine	Geneticin	4b
memTurqoise/Citrine	Reciever	memTurqoise	PB-pEF1s-Citrine (FLP-IN)	pOG44	Hygromycin	50	Monoclonal	mTurqoise2/mCitrine	Hygromycin/Geneticin	
HEK-H2B-Citrine	Reciever	Flp-In™ T-REx™ 293	CMV-TO+Cit-Int (FLP-IN)	pOG44	Hygromycin	50	Polyclonal	mCitrine	Hygromycin	6, S3, S4
HEKwt (Flp-In™ T-REx™ 293)	Reciever	HEK293						N/A		2b, 3b, 4b, 4c, 5, S1
HEK-TVA	Reciever	HEK293						N/A		2c, S1a, 5d
Gbp6	Bridge Producer	Flp-In™ T-REx™ 293	PB-pEF1s-TVAecd-Gbp6HIS-Cer-Neo	Piggybac Transposase	Geneticin	400	Polyclonal	mCerulean	Geneticin	3a, S1a
B7GG-TCer	Producer	B7GG	PB-CMVTO-TEVP-Cer-Zeo	Piggybac Transposase	Zeocin	100	Polyclonal	mCerulean	Zeocin	4, S2
B7GG	Producer	BHK21						N/A		2, 5, 6, S3, S4
EnvA-BHK	Producer	BHK21						N/A		5, 7
Gibco primary rat cortical neurons										5e, 7f, S5, S7

Supplementary Table 2: List of cell lines used for experiments and cell lines used to produce rabies viruses and bridge proteins. Highlighted blue rows indicate purchased cell lines. Producer lines are used to package the rabies virus purified for infection. Bridge producing lines are used to generate bridge protein. Sender lines are integrated with a viral glycoprotein to transmit the virus to Receiver lines in the co-culture studies. See Methods for details.
