# Supplementary material for: Engineering multiple levels of specificity in an RNA viral vector
Source: Nat Commun. 2026 Apr 2;17:4758. doi: 10.1038/s41467-026-71033-7 (PMC13216580; doi:10.1038/s41467-026-71033-7)
Supplement: Supplementary file 2 — Description of Additional Supplementary Files [file 41467_2026_71033_MOESM2_ESM.pdf]

Supplementary Movie 1: Example RVdG-P-HCVP-L infected cells with no ASV treatment (left), continuous ASV treatment (center) and ASV treatment for 6.9 days and then in media containing no ASV for 5 days (right). Top is the mCherry channel and bottom is the H2B-Citrine channel. Timestamp in the upper left corner of the mCherry channel in the first column indicates days:hours:minutes for all conditions. Yellow triangle in the upper right corner indicates ASV addition.

Supplementary Movie 2: Preliminary experiment in which example RVdG-P-HCVP-L infected cells with a single ASV treatment for 6.2 days and then in media containing no ASV for 3 days. Top is the mCherry channel and bottom is the H2B-Citrine channel. Timestamp in the upper left corner of the mCherry channel in the first column indicates days:hours:minutes for all conditions. Yellow triangle in the upper right corner indicates ASV addition. Re-emergence of the virus can be seen in the right movie.
