# Supplementary material for: Engineering multiple levels of specificity in an RNA viral vector
Source: Nat Commun. 2026 Apr 2;17:4758. doi: 10.1038/s41467-026-71033-7 (PMC13216580; doi:10.1038/s41467-026-71033-7)
Supplement: Supplementary file 3 — Reporting Summary [file 41467_2026_71033_MOESM3_ESM.pdf]

nature portfolio

Corresponding author(s): Xiaojing J. Gao and Michael B. Elowitz

Last updated by author(s): Feb 25 2026

Reporting Summary

Nature Portfolio wishes to improve the reproducibility of the work that we publish. This form provides structure for consistency and transparency in reporting. For further information on Nature Portfolio policies, see our Editorial Policies and the Editorial Policy Checklist.

Please do not complete any field with "not applicable" or n/a. Refer to the help text for what text to use if an item is not relevant to your study.
For final submission: please carefully check your responses for accuracy; you will not be able to make changes later.

Statistics

For all statistical analyses, confirm that the following items are present in the figure legend, table legend, main text, or Methods section.

n/a	Confirmed	
☐	☒	The exact sample size (*n*) for each experimental group/condition, given as a discrete number and unit of measurement
☐	☒	A statement on whether measurements were taken from distinct samples or whether the same sample was measured repeatedly
☐	☒	The statistical test(s) used AND whether they are one- or two-sided<br>*Only common tests should be described solely by name; describe more complex techniques in the Methods section.*
☒	☐	A description of all covariates tested
☐	☒	A description of any assumptions or corrections, such as tests of normality and adjustment for multiple comparisons
☐	☒	A full description of the statistical parameters including central tendency (e.g. means) or other basic estimates (e.g. regression coefficient) AND variation (e.g. standard deviation) or associated estimates of uncertainty (e.g. confidence intervals)
☐	☒	For null hypothesis testing, the test statistic (e.g. *F*, *t*, *r*) with confidence intervals, effect sizes, degrees of freedom and *P* value noted<br>*Give P values as exact values whenever suitable.*
☒	☐	For Bayesian analysis, information on the choice of priors and Markov chain Monte Carlo settings
☒	☐	For hierarchical and complex designs, identification of the appropriate level for tests and full reporting of outcomes
☒	☐	Estimates of effect sizes (e.g. Cohen's *d*, Pearson's *r*), indicating how they were calculated

Our web collection on statistics for biologists contains articles on many of the points above.

Software and code

Policy information about availability of computer code

Data collection	MATLAB 2019a or 2020a, Python, Jupyter Notebook. Flow cytometry data analysis software used for this study is available at https://antebilab.github.io/easyflow/.
Data analysis	The datasets analyzed and the computer code used during the current study are available at https://drive.google.com/drive/folders/1vccowmw-tf90eDTnrJ8AVP8WmxMcQ8d9?usp=drive_link. Prism 10.

For manuscripts utilizing custom algorithms or software that are central to the research but not yet described in published literature, software must be made available to editors and reviewers. We strongly encourage code deposition in a community repository (e.g. GitHub). See the Nature Portfolio guidelines for submitting code & software for further information.

Data

Policy information about availability of data

All manuscripts must include a data availability statement. This statement should provide the following information, where applicable:
- Accession codes, unique identifiers, or web links for publicly available datasets
- A description of any restrictions on data availability
- For clinical datasets or third party data, please ensure that the statement adheres to our policy

The datasets generated and analyzed and the computer code used during the current study are available at https://drive.google.com/drive/folders/1vccowmw-tf90eDTnrJ8AVP8WmxMcQ8d9?usp=drive_link or from the corresponding author. Flow cytometry data analysis software used for this study is available at https://antebilab.github.io/easyflow/.

Research involving human participants, their data, or biological material

Policy information about studies with human participants or human data. See also policy information about sex, gender (identity/presentation), and sexual orientation and race, ethnicity and racism.

Reporting on sex and gender	N/A
Reporting on race, ethnicity, or other socially relevant groupings	N/A
Population characteristics	N/A
Recruitment	N/A
Ethics oversight	N/A

Note that full information on the approval of the study protocol must also be provided in the manuscript.

Field-specific reporting

Please select the one below that is the best fit for your research. If you are not sure, read the appropriate sections before making your selection.

[X] Life sciences [ ] Behavioural & social sciences [ ] Ecological, evolutionary & environmental sciences

For a reference copy of the document with all sections, see nature.com/documents/nr-reporting-summary-flat.pdf

Life sciences study design

All studies must disclose on these points even when the disclosure is negative.

Sample size	Experiments were done in triplicate biological replicates. The movies are technical replicates, done in parallel.
Data exclusions	Some positions for the movies were excluded from analysis. Exclusion criteria was pre-determined: cells that were infected and remained within the field of view for five days were used for data analysis. Additionally, we excluded from analysis fields of view that were out of focus, near the edge of the well, or exhibited clumping or apoptosis within the last day of imaging.
Replication	Experiments were done multiple times over the generation of this study (non-triplicate) in the process of optimizing conditions. All HEK and primary neuron experiments were n=3 independent biological replicates.
Randomization	Viruses were passaged in parallel and all samples were done in triplicate biological replicates.
Blinding	Groups were not blinded as sample preparation followed uniform protocol. Mammalian cells were grown under identical conditions.

Behavioural & social sciences study design

All studies must disclose on these points even when the disclosure is negative.

Study description	
Research sample	
Sampling strategy	
Data collection	
Timing	
Data exclusions	
Non-participation	
Randomization	

Ecological, evolutionary & environmental sciences study design

All studies must disclose on these points even when the disclosure is negative.

Study description	
Research sample	
Sampling strategy	
Data collection	
Timing and spatial scale	
Data exclusions	
Reproducibility	
Randomization	
Blinding	

Did the study involve field work? ☐ Yes ☐ No

Field work, collection and transport

Field conditions	
Location	
Access & import/export	
Disturbance	

Reporting for specific materials, systems and methods

We require information from authors about some types of materials, experimental systems and methods used in many studies. Here, indicate whether each material, system or method listed is relevant to your study. If you are not sure if a list item applies to your research, read the appropriate section before selecting a response.

Materials & experimental systems

n/a	Involved in the study	
☐	☒	Antibodies
☐	☒	Eukaryotic cell lines
☒	☐	Palaeontology and archaeology
☒	☐	Animals and other organisms
☒	☐	Clinical data
☒	☐	Dual use research of concern
☒	☐	Plants

Methods

n/a	Involved in the study	
☐	☐	ChIP-seq
☐	☒	Flow cytometry
☐	☐	MRI-based neuroimaging

Antibodies

Antibodies used	anti-hemagglutinin antibody (HA, Abcam; catalog# ab137838) 1:500
Validation	Abcam ab137838 evaluated by immunoprecipitation, western blot, immunofluorescence, and ELISA https://www.abcam.com/en-us/products/primary-antibodies/ha-tag-antibody-ab137838

Eukaryotic cell lines

Policy information about cell lines and Sex and Gender in Research

Cell line source(s)	Flp-In T-REx 293 Cell Line (R78007) and Gibco rat cortex neurons (A1084002) were purchased from Thermo Fisher Scientific. B7GG cell line (Baby Hamster Kidney cells that contain a stably integrated T7 RNA polymerase and rabies glycoprotein) and HEK-TVA cell line (Human Embryonic Kidney cells that contain stably integrated TVA receptor) were kindly gifted from Dr. Lindsay Schwarz at St. Jude Children's Research Hospital. Derived lines will be available upon request.
Authentication	None authenticated.
Mycoplasma contamination	Cell lines were not tested for mycoplasma contamination.
Commonly misidentified lines (See ICLAC register)	HEK293. These are used in a synthetic study as engineered senders or unmodified receivers. Engineered senders undergo antibiotic selection.

Palaeontology and Archaeology

Specimen provenance	
Specimen deposition	
Dating methods	

☐ Tick this box to confirm that the raw and calibrated dates are available in the paper or in Supplementary Information.

Ethics oversight	

Note that full information on the approval of the study protocol must also be provided in the manuscript.

Animals and other research organisms

Policy information about studies involving animals; ARRIVE guidelines recommended for reporting animal research, and Sex and Gender in Research

Laboratory animals	
Wild animals	
Reporting on sex	
Field-collected samples	
Ethics oversight	

Note that full information on the approval of the study protocol must also be provided in the manuscript.

Clinical data

Policy information about clinical studies

All manuscripts should comply with the ICMJE guidelines for publication of clinical research and a completed CONSORT checklist must be included with all submissions.

Clinical trial registration	
Study protocol	
Data collection	
Outcomes	

Dual use research of concern

Policy information about dual use research of concern

Hazards

Could the accidental, deliberate or reckless misuse of agents or technologies generated in the work, or the application of information presented in the manuscript, pose a threat to:

No | Yes

- [ ] [ ] Public health
- [ ] [ ] National security
- [ ] [ ] Crops and/or livestock
- [ ] [ ] Ecosystems
- [ ] [ ] Any other significant area

Experiments of concern

Does the work involve any of these experiments of concern:

No | Yes

- [ ] [ ] Demonstrate how to render a vaccine ineffective
- [ ] [ ] Confer resistance to therapeutically useful antibiotics or antiviral agents
- [ ] [ ] Enhance the virulence of a pathogen or render a nonpathogen virulent
- [ ] [ ] Increase transmissibility of a pathogen
- [ ] [ ] Alter the host range of a pathogen
- [ ] [ ] Enable evasion of diagnostic/detection modalities
- [ ] [ ] Enable the weaponization of a biological agent or toxin
- [ ] [ ] Any other potentially harmful combination of experiments and agents

Plants

Seed stocks

Novel plant genotypes

Authentication

ChIP-seq

Data deposition

- [ ] Confirm that both raw and final processed data have been deposited in a public database such as GEO.
- [ ] Confirm that you have deposited or provided access to graph files (e.g. BED files) for the called peaks.

Data access links
May remain private before publication.

Files in database submission

Genome browser session
(e.g. UCSC)

Methodology

Replicates

Sequencing depth

Antibodies

Peak calling parameters

Data quality

Software

Flow Cytometry

Plots

Confirm that:

☐ The axis labels state the marker and fluorochrome used (e.g. CD4-FITC).

☐ The axis scales are clearly visible. Include numbers along axes only for bottom left plot of group (a 'group' is an analysis of identical markers).

☐ All plots are contour plots with outliers or pseudocolor plots.

☒ A numerical value for number of cells or percentage (with statistics) is provided.

Methodology

Sample preparation: Three days after transfection or infection, cells were prepared for flow cytometry by trypsinizing with 40-100 µL of 0.05% trypsin for 1-5 min at room temperature. Cells were then filtered through 40 µm Falcon TM Cell Strainers (Thermo Fischer Scientific, cat no. 08-771-1) and analyzed by flow cytometry.

Instrument: MACSQuant VYB, Miltenyi, CytoFLEX, Beckman Coulter, or Biorad ZE5 Cell Analyzer

Software: EasyFlow Matlab-based software package developed in-house by Yaron Antebi to process flow cytometry data.

Cell population abundance: Analyzed population events ranged from high hundreds to thousands depending on the condition. Mono-cultured cells were analyzed using FSC/SSC gating. For co-culture analysis please see Methods.

Gating strategy: The cell population was analyzed along the FSC/SSC channels. The analyzed cell population was gated as events registered above 10^5 in both the FSC/SSC channels. A manual gate was drawn around a single continuous population of which the gate excluded both the cellular debris and diagnoal streaking events. For the remainder of the analysis process, please see Methods. An example figure showing park of this gating process is shown in Fig2b.

☐ Tick this box to confirm that a figure exemplifying the gating strategy is provided in the Supplementary Information.

Magnetic resonance imaging

Experimental design

Design type

Design specifications

Behavioral performance measures

Imaging type(s)

Field strength

Sequence & imaging parameters

Area of acquisition

Diffusion MRI ☐ Used ☐ Not used

Preprocessing

Preprocessing software

Normalization

Normalization template

Noise and artifact removal

Volume censoring

Statistical modeling & inference

Model type and settings

Effect(s) tested

Specify type of analysis: ☐ Whole brain ☐ ROI-based ☐ Both

Statistic type for inference

(See Eklund et al. 2016)

Correction

Models & analysis

n/a	Involved in the study
☐	☐ Functional and/or effective connectivity
☐	☐ Graph analysis
☐	☐ Multivariate modeling or predictive analysis

Functional and/or effective connectivity

Graph analysis

Multivariate modeling and predictive analysis

This checklist template is licensed under a Creative Commons Attribution 4.0 International License, which permits use, sharing, adaptation, distribution and reproduction in any medium or format, as long as you give appropriate credit to the original author(s) and the source, provide a link to the Creative Commons license, and indicate if changes were made. The images or other third party material in this article are included in the article's Creative Commons license, unless indicated otherwise in a credit line to the material. If material is not included in the article's Creative Commons license and your intended use is not permitted by statutory regulation or exceeds the permitted use, you will need to obtain permission directly from the copyright holder. To view a copy of this license, visit http://creativecommons.org/licenses/by/4.0/
