# Supplementary material for: Engineering multiple levels of specificity in an RNA viral vector
Source: Nat Commun. 2026 Apr 2;17:4758. doi: 10.1038/s41467-026-71033-7 (PMC13216580; doi:10.1038/s41467-026-71033-7)
Supplement: Supplementary file 5 — Transparent Peer Review file [file 41467_2026_71033_MOESM5_ESM.pdf]

nature portfolio

Peer Review File

Engineering multiple levels of specificity in an RNA viral vector

Corresponding Author: Dr Michael B. Elowitz

This manuscript has been previously reviewed at another journal. This document only contains information relating to versions considered at Nature Communications.

This file contains all reviewer reports in order by version, followed by all author rebuttals in order by version.

Version 0:

Reviewer comments:

Reviewer #1

(Remarks to the Author)
The authors have done an excellent job in addressing the points we raised in our original review. We have only one suggestion: when noting ADAR-based RNA switches, it would be good to also cite Gayet et al. Nature Communications (2023). We recommend the revised paper for publication in Nature Communications.

Open Access This Peer Review File is licensed under a Creative Commons Attribution 4.0 International License, which permits use, sharing, adaptation, distribution and reproduction in any medium or format, as long as you give appropriate credit to the original author(s) and the source, provide a link to the Creative Commons license, and indicate if changes were made.
In cases where reviewers are anonymous, credit should be given to 'Anonymous Referee' and the source.
The images or other third party material in this Peer Review File are included in the article's Creative Commons license, unless indicated otherwise in a credit line to the material. If material is not included in the article's Creative Commons

license and your intended use is not permitted by statutory regulation or exceeds the permitted use, you will need to obtain permission directly from the copyright holder.
To view a copy of this license, visit https://creativecommons.org/licenses/by/4.0/
